# Analysis of genome-wide variants through bulked segregant RNA sequencing reveals a major gene for resistance to *Plasmodiophora brassicae* in *Brassica oleracea*

**DOI:** 10.1038/s41598-018-36187-5

**Published:** 2018-12-05

**Authors:** Abdulsalam Dakouri, Xingguo Zhang, Gary Peng, Kevin C. Falk, Bruce D. Gossen, Stephen E. Strelkov, Fengqun Yu

**Affiliations:** 1 Saskatoon Research and Development Centre, Agriculture and Agri-Food Canada, Saskatoon, Canada; 2 Department of Agricultural, Food and Nutritional Science, University of Alberta, Alberta, Canada; 3 The college of Agronomy, Henan Agricultural University, Nanyang, China

## Abstract

Two cabbage (*Brassica oleracea*) cultivars ‘Tekila’ and ‘Kilaherb’ were identified as resistant to several pathotypes of *Plasmodiophora brassicae*. In this study, we identified a clubroot resistance gene (*Rcr7*) in ‘Tekila’ for resistance to pathotype 3 of *P. brassicae* from a segregating population derived from ‘Tekila’ crossed with the susceptible line T010000DH3. Genetic mapping was performed by identifying the percentage of polymorphic variants (PPV), a new method proposed in this study, through bulked segregant RNA sequencing. Chromosome C7 carried the highest PPV (42%) compared to the 30–34% in the remaining chromosomes. A peak with PPV (56–73%) was found within the physical interval 41–44 Mb, which indicated that *Rcr7* might be located in this region. Kompetitive Allele-Specific PCR was used to confirm the association of *Rcr7* with SNPs in the region. *Rcr7* was flanked by two SNP markers and co-segregated with three SNP markers in the segregating population of 465 plants. Seven genes encoding TIR-NBS-LRR disease resistance proteins were identified in the target region, but only two genes, *Bo7g108760* and *Bo7g109000*, were expressed. Resistance to pathotype 5X was also mapped to the same region as *Rcr7*. *B. oleracea* lines including ‘Kilaherb’ were tested with five SNP markers for *Rcr7* and for resistance to pathotype 3; 11 of 25 lines were resistant, but ‘Kilaherb’ was the only line that carried the SNP alleles associated with *Rcr7*. The presence of *Rcr7* in ‘Kilaherb’ for resistance to both pathotypes 3 and 5X was confirmed through linkage analysis.

## Introduction

Clubroot, caused by the obligate pathogen *Plasmodiophora brassicae* Woronin, attacks several economically important members of the family *Brassicaceae*, including canola/oilseed rape (*Brassica napus* L.), broccoli and cabbage (*B. oleracea* L.)^1^. The disease has been reported in more than 60 countries, with an estimated annual yield loss globally of 15%^2^. The pathogen induces the development of characteristic clubbing symptoms on the roots of susceptible plants, which results in wilting, premature yellowing and reduced quality and yield. Clubroot was first reported on canola on the Canadian prairies in 2003^3^, and is spreading across the prairie region^4^. Crop rotation, increasing soil pH, and fungicide application^5^ have been recommended to manage clubroot on *Brassica* vegetables, but the efficacy of these methods is limited. Genetic resistance remains the most effective strategy for clubroot management^6,7^. Several pathotypes of *P. brassicae* are present in Canada (pathotypes 2, 3, 5, 5X, 6 and 8), with pathotype 3 being the most prevalent and pathotype 5X being one of the new pathotypes that can overcome the resistance in the current cohort of clubroot resistant cultivars^8–11^.

*B. napus* was originated from the interspecific hybridization of *B. rapa* and *B. oleracea*. The majority of genes for clubroot resistance that have been identified are from *B. rapa* subsp. *rapifera*^12^. Resistance in *B. rapa* is conferred by major and minor resistance genes, and resistance from *B. rapa* has been utilized widely in breeding for resistance to clubroot in *B. rapa* and *B. napus*^7,13,14^. Several clubroot resistance genes have been identified and mapped to chromosomes of *B. rapa*; *CRc*^15^ and *Rcr8*^11^ on chromosome A02, *Crr1*^16^ and *Rcr9*^11^ on chromosome A08, *Crr2*^16^ on A01, *Crr4*^16^ on A06, *Crr3*^17^, *CRa*^18^, *CRk*^15^, *CRb*^*kato*^^19^, *CRb*^20,21^, *Rcr1*^22^, *Rcr2*^23^ and *Rcr4*^11^ on chromosome A03. *CRa*, *CRb*^*kato*^ and *Crr1* have been cloned from *B. rapa*^24–26^. They encode toll-interleukin-1 receptor/nucleotide binding site/leucine-rich repeat (TIR-NBS-LRR or TNL) proteins. In contrast to *B. rapa*, no major clubroot resistance genes or lines with strong resistance have been identified in *B. oleracea*^27^.

Molecular mapping of genes in plants is based primarily on polymorphisms in genomic DNA sequences. Transcriptome analysis of short sequences generated from RNA sequencing (RNA-Seq) platforms has been used extensively for gene expression profiling and detection variants in many plant systems^28–30^.

RNA-Seq is being widely used to identify single nucleotide polymorphisms (SNPs) that can be used as molecular markers^31,32^. Also, bulk segregant analysis (BSA) has been applied for detecting molecular markers linked to traits of interest, such as disease resistance^33^. In BSA, bulks of plants with contrasting phenotype are generated. Recent studies have used bulked segregant RNA-Seq (BSR-Seq), a combination of BSA and RNA-Seq approaches^23,34–36^ to map genes of interest. Recently, BSR-Seq of genome-wide DNA variants in a *B. rapa* population were used to map clubroot resistance gene *Rcr1* onto chromosome A03. More than 70% of the variants between resistant (R) and susceptible (S) bulks were monomorphic in each chromosome except A03, where a significantly higher percentage of polymorphic variants (PPV) were present^28^. We therefore hypothesized that a gene could be genetically mapped by identifying the PPV in a genome through BSR-Seq.

A previous study identified two cabbage (*B. oleracea*) cultivars, ‘Tekila’ and ‘Kilaherb’, with strong resistance to the major strains of *P. brassicae* in western Canada^37^. The objectives of the current study were: (i) to identify resistance gene in the cabbage cultivars; (ii) to test if identification of PPV could be used for gene mapping; (iii) to characterize transcriptome-wide variation and map the resistance gene; (iv) to develop SNP markers tightly linked to the resistance gene; and (v) to examine DNA variation in the target region and identify the most probable candidates for the gene.

## Results

### Resistance to clubroot in cultivar ‘Tekila’ is controlled by one dominant gene

The resistant hybrid ‘Tekila’ was crossed with a susceptible doubled-haploid line T010000DH3 to develop an F_1_ mapping population, which is equivalent to a BC_1_ population, because the resistant parent was a hybrid. Five weeks after inoculation with pathotype 3 of *P. brassicae*, the plants were rated for clubroot symptoms. Of the 465 F_1_ plant tested, 232 plants were resistant (R) and 233 were susceptible (S). The segregation ratio was 1R: 1S (χ^2^ = 0.7, *P* = 0.40), which indicated that resistance to pathotype 3 in this cultivar was likely controlled by a single dominant gene, designated as *Rcr7* (Resistance to club-root 7).

### Sequence alignment and read mapping

Six bulks were generated; three from the R plants and three from the S plants. RNA from each individual bulk was sequenced, resulted in six RNA sequence files. The total sequence counts were over 44 million (M) for R bulks, and 41 M for the S bulks. Approximately 80% and 83% of the total counts were assembled to reference genome in R and S pools, respectively. The total length of coding sequences assigned to chromosomes in the reference genome was 62.6 Mb. The total accumulated length of coding sequences aligned to chromosomes was ~447 Mb for each of the R and S pools. This gives an estimated 7-fold depth of coverage of the gene coding portion of the reference genome^38^.

### Transcriptome haplotytping

A haplotype profile was established based on the alignment between the coding sequences of the reference genome and RNA-Seq from the R and S pools. In total, 89 haplotypes were identified, with 48 biallelic and 41 triallelic types (Supplementary Table S1). Biallelic haplotypes were the most frequent within the coding region, with a genome-wide frequency (GWF) of 145,151. In contrast, triallelic types were rare, with a GWF of 170 (Supplementary Table S1). Also, 34% of variants at triallelic sites were non-synonymous and 66% were synonymous. Chromosome C3 contained the highest number of biallelic haplotypes, with chromosome-wide haplotype frequency (CWF) of 23,906. Chromosome C6 had the lowest number, with a CWF of 11,956. Chromosome C7 had the highest number of triallelic haplotypes, with a CWF of 32, while C6 had the lowest number, with a CWF of 7 (Supplementary Table S1).

### Analysis of variants and mapping of *Rcr7* through analysis of PPV

Based on alignment with the reference genome, about 156 K variants were detected in either R or S pools, with 154 K SNPs and 2.6 K InDels in each pool (Supplementary Table S2). Also, 36% of variants per pool were nonsynonymous and 64% were synonymous (Supplementary Table S2). Overall, 36.5% of the SNPs and 51.4% of the InDels were polymorphic (Supplementary Fig. S1).

**Figure 1:**
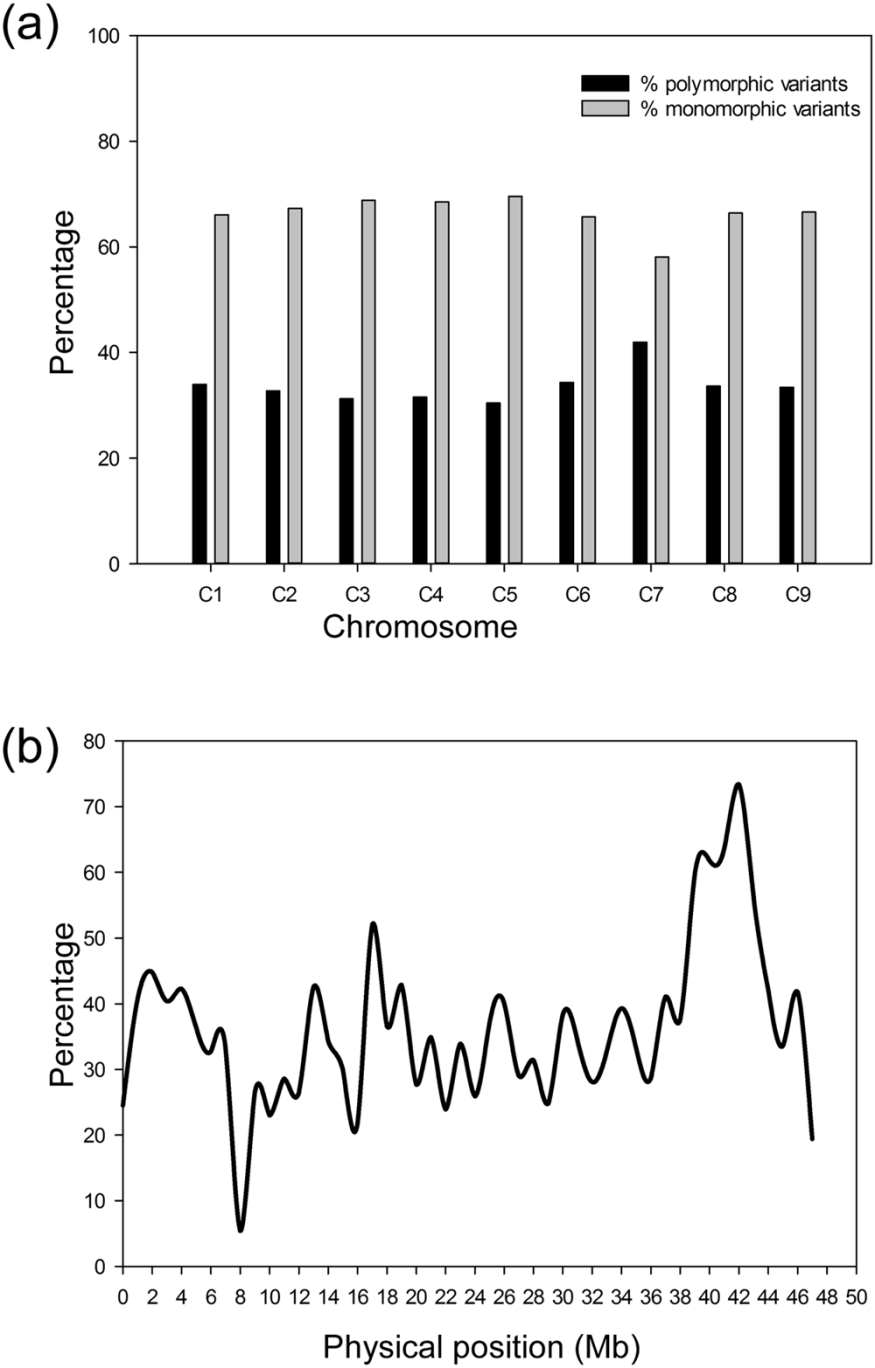
Distribution of percentage of DNA variants. (**a**) The percentage (%) of monomorphic and polymorphic variants on each chromosome. (**b**) The percentage of polymorphic variants on chromosome C7.

Chromosome C7 carried the highest PPV (42%) compared to the other eight chromosomes (30–34%) (Fig. 1a). This indicated that *Rcr7* was likely located on C7, based on PPV. In addition, a PPV peak (56–73%) was found within the physical interval 41–44 Mb (Fig. 1b), which indicated that *Rcr7* likely resided in this region of chromosome C7.

### Confirmation of *Rcr7* location and fine mapping

**Figure 2:**
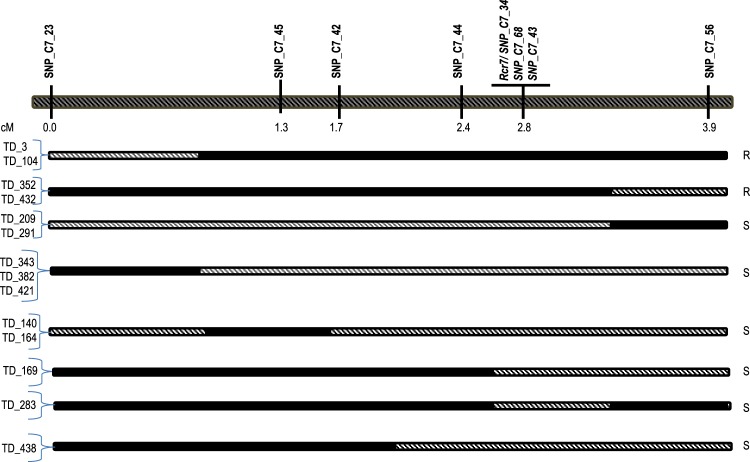
Mapping of *Rcr7* in the F_1_ population derived from the cross ‘Tekila’ × T010000DH3 with SNP markers using the KASP method. R for resistant, S for susceptible on the right, SNP markers on the top. PCR products amplified from R alleles are denoted in black and those from S alleles in hatched. The recombinants were identified from the F_1_ population consisting of 465 plants tested with pathotype 3 of *P. brassicae*.

There were 1668 SNP sites identified between physical positions 41–44 Mb of chromosome C7 where *Rcr7* was mapped using PPV (Supplementary Table S3). To fine map the gene, 465 F_1_ plants including 90R and 90S plants for RNA-Seq in the F_1_ population were analyzed with eight SNP markers at the physical position 41–44 Mb of chromosome C7 using Kompetitive Allele-Specific PCR (KASP) (Table 1, Supplementary Table S3). *Rcr7* was flanked by SNP_C7_44 and SNP_C7_56 at 0.4 and 1.1 centi-Morgan (cM) respectively, in an interval of 1.5 cM. Three SNP markers (SNP_C7_34, SNP_C7_43 and SNP_C7_68) co-segregated with *Rcr7* (Fig. 2), confirming that *Rcr7* is located at the physical position 41–44 Mb of chromosome C7.

**Table 1:** List of KASP primers, together with their physical locations and flanking sequences.

Marker ID	Physical location	Flanking sequence
SNP_C7_20	42111835	CCATGGAGGAGCTTGTGAGAATGGCTCAAACAGGTGATCCCTTGTGGGTTTCAAGCGATA[G/A]TGCGGTTGAGATTCTCAATGAGGAAGAGTATTTTCGAACGTTCCCTAGGGGAATAGGACC
SNP_C7_34	42889307	CCGGGAGTCTACGAGATAGGACTAAACTACCTTGATTTCATGGGCAAGAATAGTGGGCCT[C/T]TTTTGGACAATAAGGTTAATGTTACGAAGTGCAACATATTGGATCTCAGGTTCTGCAGAA
SNP_C7_42	42707812	CGTTCGAAGCATCATATCAAGTCCGAGGGAACCAACTTTCGGCATGTAGTTTCTCATTAT[G/A]TCGTATCTCCCCTGCACATATCACATTATATTAAGATCTCACTGCAAGATCCCACAAAAA
SNP_C7_43	42887446	TTGTTGAACTGAATCATGAAGCCATCAAGAACCGACTGCGAGTTGTTCTCGAGGAGCATA[C/T]TGTAGAACACTTGACCATCTTGTCGGGTTAATTGAGCGCTAATCTGCAGACCTTGACCGC
SNP_C7_44	42863773	CATGATCTTCGCACTTGTCTACTGTACTGCCGGAATCTCGGGAGGACACATTAACCCGGC[G/A]GTGACATTCGGTTTGTTCTTGGCGAGGAAGCTTTCTTTGACAAGAACTGTCTTCTACATA
SNP_C7_45	42581612	GATGGAACTCACAATCTCTCAGCCTGATGATTGGCATCTTCATCTCCGTGACGGCGATCT[T/C]CTTCAGGCTGTTGTTCCCCACAGGTTTATATAACTAGGACCTACAATTTACATGCATATC
SNP_C7_56	43094738	GCTTTGTTACTTGAGGCGCAAGTTAAGGATTGTTAAGGACCATAAGCTGCAGAAGAAAGG[C/A]GGTATGTCTCCGGAGAAGTTTGATTCGAGGATGGAAGAGGCTAACAACAGGCTGTCTTTC
SNP_C7_68	42888001	CAAGGTTGAAATATTTGCGAGCAGCTCATCCAGGAGTGACGGCTCAAGTTGATTTGAGTC[G/A]TCAGTAATCACAGGCTTTTCAGCTAAAACAACATCCTTCGCCGCCTAGTCAAAAGAAGAG

### Identification of variants in the target region

**Figure 3:**
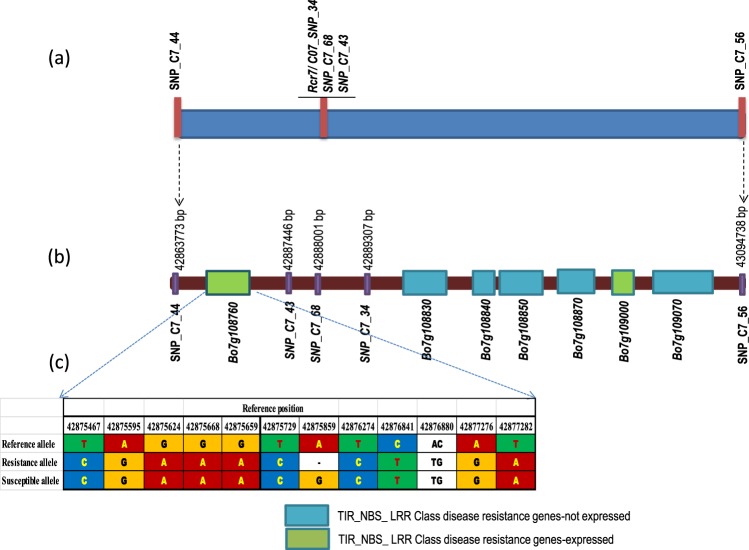
*Rcr7* location on chromosome C7. (**a**) Genetic location of *Rcr7*. (**b**) Physical locations of the SNP markers and TIR-NBS-LRR disease resistance genes in the *Rcr7* target region. The length of the boxes reflects the sizes of the genes. (**c**) Comparison of polymorphic variants at the *Bo7g108760* loci between the reference genome, R pool and S pool.

SNP_C7_44 was located in gene *Bo7g108740* encoding an Aquaporin protein at site 42,863,773 and SNP_C7_56 in gene *Bo7g109090* encoding a Receptor-like protein kinase 1-like protein at site 43,094,738 causing synonymous mutations (Fig. 3). The physical distance between these two markers was 230,966 bases. There were 36 genes, including 6 genes that encoded disease resistance proteins and 1 gene that encoded disease resistance-responsive protein in this region, based on Blast2Go search (Supplementary Table S4). Blast search at http://www.arabidopsis.org/wublast/index2.jsp indicated that all seven disease resistance genes encoded TNL proteins. The number of polymorphic variants (SNP and InDel) uniquely identified from the R bulk was further assessed in the seven genes. Five TNL genes (*Bo7g108830*, *Bo7g108840*, *Bo7g108850*, *Bo7g108870* and *Bo7g109070*) in the region did not show any expression and no short reads were assembled into the reference genome, so no polymorphic variants could be identified in the genes (Table 2). Short reads were identified in two TNL genes, *Bo7g108760* and *Bo7g109000*, in both the R and S bulks (Table 2). The coding region in *Bo7g108760* is 2724 bp in length (Table 2). One polymorphic InDel was identified (Table 2, Fig. 3). The coding region in *Bo7g109000* is 564 bp in length and no polymorphic SNPs or InDels between the R and S bulks were identified (Table 2).

**Table 2:** List of genes encoding disease resistance related proteins in the *Rcr7* C-genome target region and homologous regions in A-genome of *Brassica rapa*.

*B. oleracea*	Gene length (bp)	No. of SNP	No. of Indel	RPKM in R bulks	RPKM in S bulks	*B. rapa* homolog^b^
*Bo7g108760*	2724	4	1	2.420	2.433	*Bra019305*; A03; 25101681 to 25104380
*Bo7g108830*	1032	0	0	0.003	0.003	*Bra013698*; A01; 7228683 to 7231760
*Bo7g108840*	414	0	0	0.003	0.003	*Bra001161*; A03; 15045523 to 15048954
*Bo7g108850*	789	0	0	0.003	0.003	*Bra027889;* A09; 10044020 to 10045004
*Bo7g108870*	900	0	0	0.079	0.178	*Bra019297;* A03; 25153454 to 25154701
*Bo7g109000*	564	0	0	5.811	3.577	*Bra019277*; A03; 25319404 to 25319967
*Bo7g109070*	3009	0	0	0.237	0.160	*Bra019273*; A03; 25345715 to 25352019

^a^Each of these genes were in the TIR-NBS-LRR class, based in gene sequence blasts at http://www.arabidopsis.org/wublast/index2.jsp.

^b^Gene sequence was blasted at http://brassicadb.org/brad/blastPage.php.

The homologous regions of the A-genome of *B. rapa* corresponding to the seven TNL genes were searched. Two expressed TNL genes, *Bo7g108760* and *Bo7g109000*, as well as two unexpressed TNL genes, *Bo7g108870* and *Bo7g109070*, were homologous to the *B. rapa* genes *Bra019305*, *Bra019277*, *Bra019297* and *Bra019273* respectively. All four *B. rapa* genes reside in the 25 Mb region of chromosome A03. The unexpressed gene, *Bo7g108840*, was homologous to the *B. rapa* gene *Bra001161* in the 15 Mb region of A03; *Bo7g108830* and *Bo7g108850* were homologous to the *B. rapa* genes *Bra013698* and *Bra027889*, which are located at 7 Mb of A01 and 10 Mb of A09 respectively (Table 2).

### Genetic mapping of clubroot resistance to pathotype 3 of *P. brassicae* in ‘Kilaherb’

The two flanking markers and three co-segregating markers (Table 1) were assessed on additional 25 accessions of *B. oleracea*, including ‘Kilaherb’ (Table 3). Eleven accessions were resistant to pathotype 3 of *P. brassicae*, with 0 DSI, but only ‘Kilaherb’ carried the SNP alleles associated with *Rcr7*.

**Table 3:** Clubroot severity (disease severity index, DSI) and SNP marker profiles on accessions of *Brassica oleracea*.

Accession ID	Name	DSI	SNP_C7_ marker				
			43	34	68	44	56
CN 35413	White Flowered	78	—	—	—	—	—
Kailaan-Big Boy	Kailaan-Big Boy	44	—	—	—	—	—
CN87022	Polycaul	50	—	—	—	—	—
CGN14040	Kailan	71	—	—	—	—	—
CGN14041	Kailan	86	—	—	—	—	—
Chinese kale	Kialaan–Ii	0	—	—	—	—	—
Chinese kale	Kialaan–I	0	—	—	—	—	—
CK 1312	Nobel Jade	38	—	—	—	—	—
CHK1058	Green Jade	73	—	—	—	—	—
MU538B	UI Lan Midwater	0	—	—	—	—	—
MU550B	Green Pearl	0	—	—	—	—	—
422 C	Guy Lon	100	—	—	—	—	—
1004	1004	0	—	—	—	—	—
1005	1005	30	—	—	—	—	—
1006	1006	0	—	—	—	—	—
JL04	JL04	0	—	—	—	—	—
JL03	JL03	0	—	—	—	—	—
JL02	JL02	24	—	—	—	—	—
Jl01	Jl01	50	—	—	—	—	—
H03	H03	0	—	—	—	—	—
H02	H02	0	—	—	—	—	—
H01	H01	33	—	—	—	—	—
B04	B04	0	—	—	—	—	—
B03	B03	11	—	—	—	—	—
DH3	T010000DH3	100	—	—	—	—	—
Kilaherb	Kilaherb	0	+	+	+	+	+
Tekila	Tekila	0	+	+	+	+	+

“+” The presence of SNP allele associated with *Rcr7*; “−” absence of SNP allele associated with *Rcr7*.

**Figure 4:**
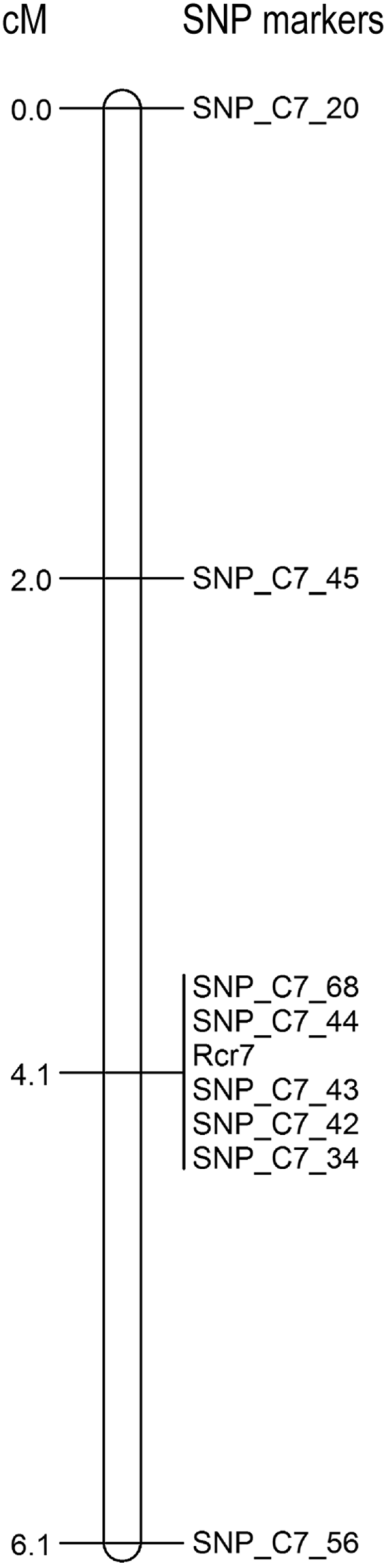
Genetic mapping of clubroot resistance in ‘Kilaherb’ × T010000DH3 F_1_ population for resistance to pathotypes 3 and 5X.

To determine if the clubroot resistance in ‘Kilaherb’ was associated with *Rcr7*, a segregating population consisting of 50 F_1_ plants from the cross ‘Kilaherb’ × T010000DH3 was assessed with the eight SNP markers identified previously. The population segregated in a 1:1 ratio (25R and 25S) to pathotype 3 of *P. brassicae*, indicating that ‘Kilaherb’ carried a single dominant clubroot resistance gene. All eight SNP markers associated with *Rcr7* in ‘Tekila’ were also associated with resistance in ‘Kilaherb’ (Fig. 4). Three SNP markers (SNP_C7_34, SNP_C7_43 and SNP_C7_68) that co-segregated with *Rcr7* also co-segregated with resistance in ‘Kilaherb’, confirming that ‘Kilaherb’ carries *Rcr7*.

### Genetic mapping of resistance to pathotype 5X of *P. brassicae* in ‘Tekila’ and ‘Kilaherb’

‘Tekila’ and ‘Kilaherb’ were resistant to pathotype 5X of *P. brassicae*. To test if the resistance to 5X was associated with *Rcr7*, a subset of the ‘Tekila’ and ‘Kilaherb’ segregating populations comprising 88 F_1_ plants each were inoculated with pathotype 5X. There were 50 R and 38S plants in the ‘Tekila’ population and 43R and 55S plants in the ‘Kilaherb’ population. Segregation for R and S in both populations was consistent with an expected ratio of 1:1 (χ^2^ = 1.64 and *P* = 0.20 in the ‘Tekila’ population and χ^2^ = 1.47 and *P* = 0.23 in the ‘Kilaherb’ population). When both populations were test with the eight SNP markers linked to *Rcr7*, all of the markers co-segregated with resistance to pathotype 5x in ‘Tekila’ and the genetic map for resistance to pathotype 5X for ‘Kilaherb’ was exactly the same as that for resistance to pathotype 3 (Fig. 4).

## Discussion

Until recently, genetic mapping to identify genetic loci governing traits of interest was time-consuming and laborious. Development of high throughput sequencing (HTS) technology has made sequencing of whole genomes comparatively quick and affordable. One application of HTS is mapping by sequencing (MBS). MBS has been used to map causal genes in the genomes of organisms such as *Arabidopsis thaliana*^3,39,40^, *Caenorhabditis elegans*^41^, wheat^35^ and maize^36^. A previous study identified a high proportion of PPV on chromosome A03 of *B. rapa* adjacent to the clubroot resistance gene *Rcr1*^28^. The current study used NGS and identification of PPV to map a major clubroot resistance gene from *B. oleracea*, which was designated as *Rcr7*. A higher PPV was identified within the physical interval 41–44 Mb of chromosome C7 in *B. oleracea*, which indicated that *Rcr7* was likely located in this region. This was confirmed via conventional mapping using linkage analysis. Taken together, these results supported the proposal that identification of PPV could be used for genetic mapping of genes of interest. However, its application and efficacy comparing with other MBS methods still needs to be determined.

Although some QTLs for clubroot resistance in *B. oleracea* have previously been identified and mapped^42^, *Rcr7* is the first major clubroot resistance gene finely mapped in the *B. oleracea* genome. Strong resistance to the main pathotypes in Canada (pathotypes 2, 3, 5, 6 and 8) had previously been identified in the A-genome resistance genes *Rcr1*, *Rcr2* and *Rc4*, but these genes did not confer resistance to pathotype 5X^11,23,43^. On the other hand, the A-genome resistance genes *Rcr8* and *Rcr9* conferred resistance to pathotype 5X, but did not confer resistance to pathotypes 2, 3, 5, 6 and 8^11^. In the current study, resistance to pathotypes 3 and 5X was associated with the *Rcr7* region. It is likely that resistance to the pathotypes in ‘Tekila’ is controlled by the single dominant gene *Rcr7* or tightly linked genes on chromosome C7. Although *Rcr7* donors ‘Takila’ and ‘Kilaherb’ also showed complete resistance to 2, 5, 6 and 8, the mapping populations were not tested against these pathotypes due to the availability of the pathotypes and the limited number of seeds in the mapping populations when we performed the study. Our previous studies on *Rcr1*, *Rcr2* and *Rc4* indicated that resistance to pathotypes 2, 3, 5. 6 and 8 was associated^11,23,28^. Further studies are needed to determine if resistance to these pathotypes is associated with *Rcr7*.

When a set of *B. oleracea* lines were tested for resistance to pathotype 3 and assessed using the flanking and co-segregating markers associated with *Rcr7*, ‘Kilaherb’ had the same phenotype and marker profile at all of the marker loci as ‘Tekila’. Segregation analysis of the ‘Kilaherb’ × T010000DH3 F_1_ population indicated that resistance was controlled by a major dominant resistance gene, similarly to ‘Tekila’. Marker profiling for a set of F_1_ segregating lines also supported the hypothesis that ‘Kilaherb’ carried *Rcr7*. Both ‘Tekila’ and ‘Kilaherb’ were developed by Syngenta, so it is possible that the same source of resistance was used in both hybrids.

A patent (http://www.google.com/patents/EP1525317A1?cl=en) on “Clubroot Resistant *Brassica oleracea* Plants” shows that Syngenta developed a monogenic dominant resistance to the disease clubroot introgressed from *B. rapa*. Therefore, it is possible that *Rcr7* was originated from A-genome of *B. rapa*. Research in Canada has focused on identification of clubroot resistance genes in *B. rapa*^11,22,23,28,44^ and the A-genome of *B. napus*^45–47^. Most of the clubroot resistance genes effective against pathotype 3 were mapped into the 24 Mb region of chromosome A03 that contains the cloned clubroot resistance genes *CRa/CRb*^*kato*^, where a cluster of four TNL genes, *Bra019413*, *Bra019412*, *Bra019410* and *Bra019409* are located. However, the genes/alleles do not confer resistance to pathotype 5X^11,23,28^, while resistance to both pathotypes 3 and 5X was associated with *Rcr7*. A search of the homologous regions of A-genome corresponding to the seven TNL genes in the *Rcr7* target region of chromosome C7 revealed that the homologous genes in chromosome A03 were not in the previous mapped A03 genes (*Rcr1/Rcr2/Rcr4/CRa/CRb*) region. In addition, gene specific SNP markers associated with *Rcr1*/*Rcr2/Rcr4*^11,23,28^ in the F_1_ population of ‘Tekila’ × T010000DH3 were either monomorphic or not tightly linked to *Rcr7* (data not shown). Therefore, *Rcr7* is a clubroot resistance gene mapped in the *B. oleracea* genome, possibly originating from a gene in chromosome A03 of *B. rapa*, but different from *Rcr1/Rcr2*/*Rcr4* based on the genetic location and resistance specificity. However, the *Rcr7* origin and clubroot genes corresponding to *Rcr7* in *B. rapa* require further investigations.

Only 2 of the 7 TNL genes associated with *Rcr7* (*Bo7g108760* and *Bo7g109000*) were expressed in this study. No polymorphic variants were identified in *Bo7g109000*, but one polymorphic InDel was found in *Bo7g108760*. Therefore, *Bo7g108760* is possibly a candidate of *Rcr7*. However, the profiles of gene expression and DNA variants were not fully captured in this study due to low depth of sequencing, so other candidates may exist. The identity of the TNL gene corresponding to *Rcr7* will be addressed after the gene has been cloned.

The transition forms (C → T, G → A) dominated single-base substitutions with the ratio of 1.5 (transition): 1 (tranversion). This ratio was similar to the 2:1 ratio reported from the human genome^48,49^. Despite the bialleleic nature of most SNPs in the current analysis, some loci with three segregating nucleotides may also exist in certain populations^50^. Triallelic SNPs were previously reported in the human genome at a very low frequency compared to biallelic variants. Human transcriptome analysis showed that triallelic sites would likely cause non-synonymous^48^ changes. In the current study, 34% of the triallelic SNPs resulted in non-synonymous variation in the *B. oleracea* reference genome.

## Materials and Methods

Schematic flowchart of the experimental procedure is shown in Supplementary Fig. S2.

### Plant mapping population

The study assessed F_1_ populations derived from crosses of two clubroot- resistant hybrid cabbage cultivars, ‘Tekila’ and ‘Kilaherb’ (Syngenta Canada), with a clubroot- susceptible doubled haploid (DH) line T010000DH3 derived from Chinese kale (*B. oleracea*) cultivar ‘TO1434’ (Saskatoon Research and Development Centre, Saskatoon, SK, Canada). ‘Tekila’ and ‘Kilaherb’ were completely resistant to pathotypes 2, 3, 5, 6 and 8 of *P. brassicae* in an initial screening^37^ and strains of pathotype 5X (F. Yu unpublished data). ‘Tekila’ and ‘Kilaherb’ were vernalized and crossed with T010000DH3. Also, 24 accessions of *B. oleracea* were assessed in this study (Table 3).

### Evaluation of plants for resistance to clubroot

A field population of *P. brassicae* identified as pathotype 3^22^ and a field population L-G02 of pathotype 5X^10^ were used for inoculation in this study. Clubroot reaction was assessed in controlled environment studies, as described by Chu, *et al*.^22^. Briefly, the plants were assessed for clubroot symptoms at 5 weeks after seeding using a 0 to 3 scale, where 0 = no symptoms and 3 = large galls^51^. A clubroot rating of 0 was defined as R, 1–3 as S. A disease severity index (DSI)^51,52^ was computed based on 7-14 plants of each of the *B. oleracea* accessions.

### RNA sequencing, read mapping, variant analysis and gene annotation

At 12 days after seeding, leaf tissue was collected from each F_1_ plant for RNA extraction. The leaf tissue of each individual plant was stored at −80 °C until after its clubroot reaction had been assessed. Then the F_1_ plants were separated into resistant and susceptible pools. From each pool, three bulks were randomly generated, with each bulk containing 30 plants. RNA was extracted from each bulk using Qiagen RNA extraction kits as per manufacturer’s instruction (Qiagen, Toronto, ON). A cDNA library for each bulk was constructed following Illumina TruSeq® RNA Sample Preparation v2 Guide (RS-122-9001DOC, Illumina Inc.; San Diego, CA). Sequencing samples were prepared using MiSeq® Reagent Kit V3, as per the manufacturer’s instruction (MS-102-3001, Illumina Inc.). Sequencing was performed at the University of Saskatchewan, Saskatoon, SK using an Illuimna MiSeq® System (Illumina Inc.) with 75 cycles, which resulted in 75 bp pair-end reads. The short sequence reads of the pooled resistance bulks and pooled susceptible bulks were mapped to the nine chromosomes (C1 to C9) of *B. oleracea* reference genome v2.1^38^ following the pooled sample assembly method described by^28^ using SeqMan NextGen in DNASTAR.12 software (Lasergene Inc., Madison, WI). For variant analysis, pooled sample assembly (PSA) was selected because it tends to produce a more complete sequence coverage compared to single-sample assembly^28^.

Variants (SNPs and InDels) analysis was performed using SeqMan Pro 12 in DNASTAR.12 software, utilizing the following parameters: SNP% ≥15%, P not ref ≥50%, Q-call ≥15 and depth ≥5.

Gene annotation was analyzed with Blast2GO^53^. Further confirmation of the genes with TNL domains was performed with *Arabidopsis thaliana* WU-BLAST2 Search at http://www.arabidopsis.org/wublast/index2.jsp.

### KASP assay and linkage analysis

The sequences flanking the selected SNP sites were used for designing KASP primers (Table 1). DNA oligoes were synthesized at Integrated DNA Technologies Inc. (Coralville, IA, USA). The primers carry standard FAM or HEX tails (FAM tail: 5′GAAGGTGACCAAGTTCATGCT3′; hex TAIL 5′GAAGGTCGGAGTCAACGGATT3′) with the targeted SNP at the 3′ end. KASP assays were performed following the principle and procedure available at http://www.kbioscience.co.uk/reagents/KASP_manual.pdf and http://www.kbioscience.co.uk/download/KASP.swf. The assays were performed using a StepOne Plus real time qPCR system (Applied Biosystem, Mississauga, ON). Linkage analysis was performed using JoinMap V.4^54^.

## Electronic supplementary material

Supplementary Tables and Figures

## References

[1] Karling, J. S. The Plasmodiophorales 2nd ed. Hafner Publishing Company, Inc., New York (1968).

[2] Dixon GR (2009). The occurrence and economic impact of *Plasmodiophora brassicae* and clubroot disease. J. Plant Growth Regul..

[3] Tewari JP (2005). Identification of clubroot of crucifers on canola *(Brassica napu*s) in Alberta. Plant Dis..

[4] Gossen BD (2015). Spread of clubroot on canola in Canada, 2003–2014. Old pathogen, new home. Can. J. Plant Pathol..

[5] Donald EC, Porter I (2009). Integrated control of clubroot. J. Plant Growth Regul..

[6] Voorrips RE (1995). *Plasmodiophora brassicae*: Aspects of pathogenesis and resistance in *Brassica oleracea*. Euphytica..

[7] Diederichsen E, Frauen M, Linders E, Hatakeyama K, Hirai M (2009). Status and perspectives of clubroot resistance breeding in crucifer crops. J. Plant Growth Regul..

[8] Xue C, Cao T, Howard RJ, Hwang SF, Strelkov SE (2008). Isolation and variation in virulence of single-spore isolates of *Plasmodiophora brassicae* from Canada. Plant Dis..

[9] Strelkov SE (2008). Incidence of clubroot in Alberta in 2007. Can. Plant Dis. Surv..

[10] Strelkov SE, Hwang SF, Manolii VP, Cao T, Feindel D (2016). Emergence of new virulence phenotypes of *Plasmodiophora brassicae* on canola (*Brassica napus*) in Alberta, Canada. Eur. J. Plant Pathol..

[11] Yu, F. *et al*. Genotyping-by-sequencing reveals three QTL for clubroot resistance to six pathotypes of *Plasmodiophora brassicae* In *Brassica rapa. Scientific* Reports (in press) (2017). 10.1038/s41598-017-04903-2 PMC5495781 28674416

[12] Buczacki ST (1975). Study of physiologic specialization in Plasmodiophora brassicae: Proposals for attempted rationalization through an international approach. Trans. Br. Mycol. Soc..

[13] Gustafsson M, Falt AS (1986). Genetic studies on resistance to clubroot in *Brassica napus*. Ann. Appl. Biol..

[14] Diederichsen E, Beckmann J, Schondelmeier J, Dreyer F (2006). Genetics of clubroot resistance in *Brassica napus* ‘Mendel’. Acta Hort..

[15] Sakamoto K, Saito A, Hayashida N, Taguchi G, Matsumoto E (2008). Mapping of isolate-specific QTLs for clubroot resistance in Chinese cabbage (*Brassica rapa* L. ssp. pekinensis). Theor. Appl. Genet..

[16] Suwabe K (2003). Identification of two loci for resistance to clubroot (*Plasmodiophora brassicae* Woronin) In *Brassica rapa L*. Theor. Appl. Genet..

[17] Saito M (2006). Fine mapping of the clubroot resistance gene Crr3 in *Brassica rapa*. Theor. Appl. Genet..

[18] Hayashida N (2008). Construction of a practical SCAR marker linked to clubroot resistance in Chinese cabbage, with intensive analysis of HC352b genes. Engei. Gakkai. Zasshi..

[19] Kato T, Hatakeyama K, Fukino N, Matsumoto S (2013). Fine mapping of the clubroot resistance gene CRb and development of a useful selectable marker in *Brassica rapa*. Breed. Sci..

[20] Piao ZY, Deng YQ, Choi SR, Park YJ, Lim YP (2004). SCAR and CAPS mapping of *CRb*, a gene conferring resistance to *Plasmodiophora brassicae* in Chinese cabbage (*Brassica rapa* ssp. pekinensis). Theor. Appl. Genet..

[21] Zhang T (2014). Fine genetic and physical mapping of the *CRb* gene conferring resistance to clubroot disease in *Brassica rapa*. Mol. Breed..

[22] Chu M (2014). Fine mapping of Rcr1 and analyses of its effect on transcriptome patterns during infection by Plasmodiophora brassicae. BMC Genomics..

[23] Huang, Z. *et al*. Fine Mapping of a Clubroot Resistance Gene in Chinese Cabbage Using SNP Markers Identified from Bulked Segregant RNA Sequencing. *Front Plant Sci*. **8**, 10.3389/fpls.2017.01448 (2017). 10.3389/fpls.2017.01448 PMC5581393 28894454

[24] Hatakeyama K (2013). Identification and characterization of Crr1a, a gene for resistance to clubroot disease (*Plasmodiophora brassicae* Woronin) In *Brassica rapa L*. PLoS ONE..

[25] Hatakeyama K (2017). The tandem repeated organization of NB-LRR genes in the clubroot-resistant CRb locus in *Brassica rapa* L. Mol. Genet. Genomics..

[26] Ueno H (2012). Molecular characterization of the CRa gene conferring clubroot resistance in *Brassica rapa*. Plant Mol. Biol..

[27] Piao Z, Ramchiary N, Lim YP (2009). Genetics of clubroot resistance in Brassica species. J. Plant Growth Regul..

[28] Yu F (2016). Identification of genome-wide variants and discovery of variants associated with *Brassica rapa* clubroot resistance gene Rcr1 through bulked segregant RNA sequencing. PLoS ONE..

[29] Barbazuk WB, Emrich SJ, Chen HD, Li L, Schnable PS (2007). SNP discovery via 454 transcriptome sequencing. Plant J..

[30] Chepelev I, Wei G, Tang Q, Zhao K (2009). Detection of single nucleotide variations in expressed exons of the human genome using RNA-Seq. Nucleic Acids Res..

[31] Michelmore RW, Paran I, Kesseli RV (1991). Identification of markers linked to disease-resistance genes by bulked segregant analysis: a rapid method to detect markers in specific genomic regions by using segregating populations. Proc. Natl Acad. Sci. USA.

[32] Ramirez-Gonzalez, R. H. *et al*. RNA-Seq bulked segregant analysis enables the identification of high-resolution genetic markers for breeding in hexaploid wheat. *Plant Biotechnol. J*. **13**, 613–624. 10.1111/pbi.12281 25382230

[33] Trick M (2012). Combining SNP discovery from next-generation sequencing data with bulked segregant analysis (BSA) to fine-map genes in polyploid wheat. BMC Plant Biol..

[34] Liu S, Yeh CT, Tang HM, Nettleton D, Schnable PS (2012). Gene mapping via bulked segregant RNA-Seq (BSR-Seq). PLoS ONE..

[35] Du, H. *et al*. Bulked Segregant RNA-seq Reveals Differential Expression and SNPs of Candidate Genes Associated with Waterlogging Tolerance in Maize. *Frontiers in plant science* **8**, 1022, 10.3389/fpls.2017.01022 (2017). 10.3389/fpls.2017.01022 PMC5470080 28659961

[36] Austin RS (2011). Next-generation mapping of Arabidopsis genes. Plant J..

[37] Peng, G. *et al*. Sources of resistance to *Plasmodiophora brassicae* (clubroot) pathotypes virulent on canola. *Can. J. Plant Pathol.* **36**, 89–99, 10.1080/07060661.2013.863805 (2014).

[38] Parkin IA (2014). Transcriptome and methylome profiling reveals relics of genome dominance in the mesopolyploid *Brassica oleracea*. Gen. Biol..

[39] Mokry M (2011). Identification of factors required for meristem function in Arabidopsis using a novel next generation sequencing fast forward genetics approach. BMC Genomics..

[40] Schneeberger K (2009). SHOREmap: simultaneous mapping and mutation identification by deep sequencing. Nat. Methods..

[41] Zuryn S, Le Gras S, Jamet K, Jarriault S (2010). A Strategy for Direct Mapping and Identification of Mutation by Whole-Genome Sequencing. Genetics..

[42] Crute IR, Gray AR, Crisp P, Buczacki ST (1980). Variation in *Plasmodiophora brassicae* and resistance to clubroot disease in brassica and allied crops: A critical review. Plant Breed..

[43] Yu J (2014). Genome-wide comparative analysis of NBS-encoding genes between Brassica species and *Arabidopsis thaliana*. BMC Genomics..

[44] Gao F, Hirani AH, Liu J (2014). Fine mapping a clubroot resistance locus in Chinese cabbage. J. Amer. Soc. Hort. Sci..

[45] Fredua-Agyeman R, Rahman MH (2016). Mapping of the clubroot disease resistance in spring *Brassica napus* canola introgressed from European winter canola cv. ‘Mendel’. Euphytica..

[46] Hasan MJ, Rahman H (2016). Genetics and molecular mapping of resistance to *Plasmodiophora brassicae* pathotypes 2, 3, 5, 6, and 8 in rutabaga (*Brassica napus* var. napobrassica). Genome..

[47] Zhang H (2016). Mapping of clubroot (Plasmodiophora brassicae) resistance in canola (*Brassica napus*). Plant Pathol..

[48] Cao M (2015). Analysis of human triallelic SNPs by next-generation sequencing. Ann. Hum. Genet..

[49] Wang DG (1998). Large-scale identification, mapping, and genotyping of single-nucleotide polymorphisms in the human genome. Science..

[50] Hodgkinson A, Eyre-Walker A (2010). Human triallelic sites: Evidence for a new mutational mechanism?. Genetics..

[51] Strelkov SE, Tewari JP, Smith-Degenhardt E (2006). Characterization of *Plasmodiophora brassicae* populations from Alberta. Canada. Can. J. Plant Pathol..

[52] Crête R, Laliberté J, Jasmin JJ (1963). Lutte chimique contre la hernie, *Plasmodiophora brassicae* Wor., des cruciferes en sols mineral et organique. Can. J. Plant. Sci..

[53] Conesa, A. *et al*. Blast2GO: A universal tool for annotation, visualization and analysis in functional genomics research. *Bioinformatics*. **21**, 3674–3676 (2005). 10.1093/bioinformatics/bti610 16081474

[54] Van Ooijen JW, Voorrips RE (2001). JoinMap 3.0: Software for the calculation of genetic linkage maps. Plant Research International, Wageningen, Netherlands. Stam (1993). The Plant Journal..

