# Supplementary material for: Analysis of genome-wide variants through bulked segregant RNA sequencing reveals a major gene for resistance to Plasmodiophora brassicae in Brassica oleracea
Source: Sci Rep. 2018 Dec 5;8:17657. doi: 10.1038/s41598-018-36187-5 (PMC6281628; doi:10.1038/s41598-018-36187-5)
Supplement: Supplementary file 1 — Supplementary Tables and Figures [file 41598_2018_36187_MOESM1_ESM.docx]

**Analysis of genome-wide variants through bulked segregant RNA sequencing reveals a major gene for resistance to *Plasmodiophora brassicae* in *Brassica oleracea***

Abdulsalam Dakouri^1^, Xingguo Zhang^1,3^ , Gary Peng^1^ , Kevin C. Falk^1^, Bruce D. Gossen^1^, Stephen E. Strelkov^2^, Fengqun Yu^1,^*

^1^Saskatoon Research and Development Centre, Agriculture and Agri-Food Canada, Canada

^2^Department of Agricultural, Food and Nutritional Science, University of Alberta, Canada

^3^The college of Agronomy, Henan Agricultural University, China

*Correspondence and requests for materials should be addressed to F.Y. (email: [fengqun.yu@agr.gc.ca](mailto:fengqun.yu@agr.gc.ca))

**Table S1.** **Haplotype analysis of transcriptome, chromosome and genome wide haplotype frequencies**

| Group |  | Haplotype |  | Chromosome wide haplotype frequency (CWF) | | | | | | | |  | Transcriptome wide haplotye  frequency (GWF) |
| --- | --- | --- | --- | --- | --- | --- | --- | --- | --- | --- | --- | --- | --- |
|  | Reference | R-Pool | S-Pool | C1 | C2 | C3 | C4 | C5 | C6 | C7 | C8 | C9 |  |
| Biallelic haplotye | A | C | C | 724 | 672 | 1076 | 766 | 851 | 566 | 718 | 690 | 844 | 6907 |
|  | A | G | G | 2122 | 1908 | 3335 | 2194 | 2393 | 1604 | 2106 | 1988 | 2443 | 20093 |
|  | A | T | T | 706 | 662 | 1159 | 780 | 806 | 492 | 679 | 653 | 819 | 6756 |
|  | C | A | A | 733 | 620 | 1166 | 723 | 809 | 509 | 715 | 646 | 766 | 6687 |
|  | C | G | G | 651 | 601 | 1059 | 670 | 755 | 524 | 680 | 693 | 732 | 6365 |
|  | C | T | T | 2125 | 1910 | 3293 | 2070 | 2336 | 1558 | 3320 | 1866 | 2351 | 20829 |
|  | G | A | A | 2029 | 1869 | 3214 | 2232 | 2418 | 1642 | 2023 | 1870 | 2280 | 19577 |
|  | G | C | C | 641 | 580 | 1039 | 646 | 781 | 495 | 662 | 626 | 764 | 6234 |
|  | G | T | T | 703 | 640 | 1102 | 705 | 822 | 561 | 748 | 656 | 839 | 6776 |
|  | T | A | A | 701 | 651 | 1143 | 708 | 821 | 561 | 664 | 642 | 794 | 6685 |
|  | T | C | C | 2098 | 1815 | 3301 | 2207 | 2278 | 1575 | 2024 | 1929 | 2364 | 19591 |
|  | T | G | G | 701 | 640 | 1197 | 720 | 841 | 595 | 654 | 643 | 797 | 6788 |
|  | A | A | C | 25 | 29 | 41 | 34 | 25 | 21 | 28 | 21 | 40 | 264 |
|  | A | A | G | 98 | 69 | 118 | 75 | 93 | 68 | 81 | 62 | 106 | 770 |
|  | A | A | T | 31 | 25 | 46 | 24 | 27 | 24 | 26 | 30 | 30 | 263 |
|  | C | C | A | 29 | 29 | 47 | 23 | 28 | 21 | 36 | 28 | 37 | 278 |
|  | C | C | G | 29 | 25 | 47 | 29 | 30 | 29 | 38 | 25 | 29 | 281 |
|  | C | C | T | 68 | 61 | 116 | 60 | 87 | 59 | 85 | 90 | 91 | 717 |
| Biallelic haplotye | G | G | A | 93 | 70 | 119 | 88 | 88 | 73 | 83 | 86 | 96 | 796 |
|  | G | G | C | 35 | 27 | 36 | 21 | 29 | 17 | 26 | 24 | 41 | 256 |
|  | G | G | T | 39 | 27 | 43 | 24 | 35 | 27 | 36 | 28 | 27 | 286 |
|  | T | T | A | 34 | 21 | 36 | 26 | 29 | 19 | 31 | 20 | 32 | 248 |
|  | T | T | C | 83 | 79 | 127 | 91 | 62 | 56 | 81 | 81 | 64 | 724 |
|  | T | T | G | 13 | 30 | 41 | 34 | 23 | 30 | 24 | 36 | 27 | 258 |
|  | A | C | A | 26 | 22 | 36 | 21 | 18 | 24 | 27 | 19 | 22 | 215 |
|  | A | G | A | 63 | 51 | 94 | 63 | 62 | 49 | 106 | 68 | 81 | 637 |
|  | A | T | A | 24 | 21 | 26 | 27 | 22 | 20 | 39 | 15 | 29 | 223 |
|  | C | A | C | 31 | 25 | 35 | 26 | 29 | 22 | 33 | 15 | 25 | 241 |
|  | C | G | C | 21 | 24 | 17 | 20 | 21 | 13 | 28 | 18 | 27 | 189 |
|  | C | T | C | 70 | 70 | 104 | 64 | 67 | 56 | 106 | 65 | 73 | 675 |
|  | G | A | G | 65 | 60 | 103 | 77 | 82 | 53 | 98 | 69 | 73 | 680 |
|  | G | C | G | 27 | 22 | 33 | 25 | 29 | 17 | 24 | 23 | 31 | 231 |
|  | G | T | G | 24 | 28 | 43 | 26 | 36 | 22 | 29 | 23 | 23 | 254 |
|  | T | A | T | 25 | 21 | 31 | 24 | 28 | 13 | 36 | 19 | 26 | 223 |
|  | T | C | T | 64 | 64 | 91 | 77 | 81 | 55 | 97 | 54 | 89 | 672 |
|  | T | G | T | 20 | 21 | 29 | 29 | 28 | 21 | 37 | 30 | 34 | 249 |
|  | A | InDel | InDel | 33 | 42 | 77 | 47 | 56 | 35 | 44 | 46 | 52 | 432 |
| Biallelic haplotye | C | InDel | InDel | 48 | 59 | 85 | 55 | 61 | 35 | 55 | 41 | 63 | 502 |
|  | G | InDel | InDel | 68 | 54 | 80 | 61 | 64 | 37 | 48 | 61 | 71 | 544 |
|  | T | InDel | InDel | 62 | 66 | 89 | 47 | 70 | 47 | 64 | 55 | 69 | 569 |
|  | A | InDel | A | 1 | 2 | 0 | 1 | 2 | 1 | 1 | 1 | 2 | 11 |
|  | C | InDel | C | 2 | 2 | 4 | 1 | 2 | 1 | 3 | 3 | 2 | 20 |
|  | G | InDel | G | 1 | 1 | 3 | 4 | 3 | 3 | 1 | 0 | 2 | 18 |
|  | T | InDel | T | 4 | 2 | 2 | 2 | 5 | 2 | 5 | 2 | 1 | 25 |
|  | A | A | InDel | 0 | 5 | 7 | 0 | 1 | 2 | 6 | 3 | 4 | 28 |
|  | C | C | InDel | 3 | 2 | 5 | 3 | 2 | 2 | 6 | 8 | 2 | 33 |
|  | G | G | InDel | 2 | 1 | 5 | 0 | 2 | 1 | 1 | 4 | 6 | 22 |
|  | T | T | InDel | 3 | 3 | 6 | 3 | 3 | 2 | 3 | 5 | 1 | 29 |
| Total |  |  |  | 15198 | 13728 | 23906 | 15653 | 17241 | 11659 | 16465 | 14080 | 17221 | 145151 |
| Triallelic haplotye | A | C | G | 1 | 0 | 0 | 0 | 0 | 1 | 1 | 1 | 0 | 4 |
|  | A | C | T | 0 | 0 | 0 | 0 | 1 | 1 | 1 | 0 | 0 | 3 |
|  | A | G | C | 0 | 0 | 1 | 2 | 0 | 1 | 2 | 0 | 0 | 6 |
|  | A | G | T | 1 | 0 | 1 | 0 | 1 | 0 | 3 | 1 | 1 | 8 |
|  | A | T | C | 2 | 0 | 2 | 2 | 0 | 0 | 3 | 1 | 1 | 11 |
|  | A | T | G | 0 | 0 | 2 | 0 | 0 | 0 | 0 | 2 | 1 | 5 |
|  | C | A | G | 1 | 0 | 2 | 0 | 0 | 0 | 0 | 0 | 0 | 3 |
| Triallelic haplotye | C | A | T | 0 | 0 | 0 | 0 | 0 | 0 | 1 | 1 | 0 | 2 |
|  | C | G | A | 0 | 0 | 0 | 0 | 0 | 0 | 1 | 0 | 1 | 2 |
|  | C | G | T | 1 | 2 | 0 | 0 | 0 | 0 | 0 | 0 | 0 | 3 |
|  | C | T | A | 2 | 2 | 0 | 0 | 1 | 0 | 2 | 0 | 1 | 8 |
|  | C | T | G | 0 | 0 | 1 | 0 | 0 | 0 | 0 | 0 | 0 | 1 |
|  | G | A | C | 0 | 0 | 0 | 0 | 1 | 2 | 1 | 0 | 1 | 5 |
|  | G | A | T | 1 | 0 | 1 | 1 | 1 | 0 | 2 | 1 | 1 | 8 |
|  | G | C | A | 1 | 1 | 3 | 1 | 0 | 0 | 0 | 0 | 0 | 6 |
|  | G | C | T | 1 | 1 | 1 | 0 | 0 | 0 | 1 | 0 | 0 | 4 |
|  | G | T | A | 0 | 2 | 2 | 0 | 1 | 1 | 0 | 0 | 0 | 6 |
|  | G | T | C | 2 | 2 | 0 | 0 | 2 | 0 | 1 | 1 | 1 | 9 |
|  | T | A | C | 0 | 2 | 1 | 2 | 1 | 0 | 0 | 0 | 0 | 6 |
|  | T | A | G | 0 | 0 | 0 | 2 | 2 | 0 | 0 | 2 | 1 | 7 |
|  | T | C | A | 1 | 0 | 2 | 3 | 0 | 0 | 3 | 1 | 2 | 12 |
|  | T | C | G | 0 | 0 | 0 | 0 | 0 | 0 | 2 | 0 | 0 | 2 |
|  | T | G | A | 0 | 1 | 1 | 2 | 1 | 0 | 3 | 0 | 1 | 9 |
|  | T | G | C | 2 | 0 | 1 | 1 | 0 | 0 | 2 | 0 | 0 | 6 |
|  | A | C | InDel | 0 | 0 | 0 | 0 | 1 | 0 | 0 | 0 | 0 | 1 |
|  | A | G | InDel | 0 | 0 | 0 | 0 | 0 | 0 | 1 | 1 | 0 | 2 |
| Triallelic haplotye | A | T | InDel | 0 | 0 | 0 | 1 | 0 | 0 | 0 | 0 | 0 | 1 |
|  | C | T | InDel | 0 | 0 | 4 | 0 | 0 | 0 | 0 | 0 | 2 | 6 |
|  | C | G | InDel | 1 | 0 | 1 | 0 | 0 | 0 | 0 | 0 | 0 | 2 |
|  | G | C | InDel | 0 | 0 | 0 | 0 | 1 | 0 | 0 | 0 | 1 | 2 |
|  | G | T | InDel | 0 | 0 | 1 | 0 | 0 | 0 | 0 | 0 | 0 | 1 |
|  | T | A | InDel | 0 | 0 | 1 | 0 | 0 | 0 | 0 | 0 | 0 | 1 |
|  | T | C | InDel | 1 | 0 | 0 | 0 | 0 | 0 | 1 | 0 | 0 | 2 |
|  | T | G | InDel | 0 | 0 | 0 | 1 | 0 | 1 | 0 | 0 | 0 | 2 |
|  | A | InDel | C | 0 | 0 | 1 | 0 | 0 | 0 | 0 | 0 | 0 | 1 |
|  | A | InDel | G | 0 | 2 | 0 | 0 | 1 | 0 | 0 | 0 | 0 | 3 |
|  | C | InDel | T | 1 | 0 | 0 | 0 | 0 | 0 | 0 | 0 | 0 | 1 |
|  | G | InDel | A | 0 | 0 | 1 | 0 | 0 | 0 | 1 | 1 | 0 | 3 |
|  | G | InDel | T | 0 | 0 | 0 | 0 | 1 | 0 | 0 | 0 | 0 | 1 |
|  | T | InDel | A | 0 | 1 | 0 | 1 | 0 | 0 | 0 | 0 | 0 | 2 |
|  | T | InDel | C | 2 | 0 | 1 | 0 | 0 | 0 | 0 | 0 | 0 | 3 |
| Total |  |  |  | 21 | 16 | 31 | 19 | 16 | 7 | 32 | 13 | 15 | 170 |

# Table S2. Total variants, average per Kb, their impacts and types

| Chromosome | Total Variants | | Average per Kb | | Synonymous | | Non-synonymous | | SNPs | | Indels | |
| --- | --- | --- | --- | --- | --- | --- | --- | --- | --- | --- | --- | --- |
|  | R | S | R | S | R | S | R | S | R | S | R | S |
| C1 | 16305 | 16452 | 2.52 | 2.54 | 10571 | 10661 | 5734 | 5791 | 16037 | 16179 | 268 | 273 |
| C2 | 14679 | 14678 | 2.51 | 2.51 | 9342 | 9111 | 5337 | 5567 | 14421 | 14414 | 258 | 264 |
| C3 | 25318 | 25804 | 2.54 | 2.59 | 16124 | 16380 | 9194 | 9424 | 24926 | 25381 | 392 | 423 |
| C4 | 16511 | 16737 | 2.50 | 2.54 | 10824 | 10874 | 5687 | 5863 | 16265 | 16469 | 246 | 268 |
| C5 | 18156 | 18333 | 3.12 | 3.15 | 11416 | 11495 | 6740 | 6838 | 17847 | 18026 | 309 | 307 |
| C6 | 12423 | 12644 | 2.02 | 2.06 | 7854 | 8005 | 4569 | 4639 | 12225 | 12453 | 198 | 191 |
| C7 | 19296 | 17745 | 2.57 | 2.36 | 12520 | 11363 | 6776 | 6382 | 19012 | 17465 | 284 | 280 |
| C8 | 14964 | 15163 | 2.02 | 2.05 | 9484 | 9570 | 5480 | 5593 | 14705 | 14897 | 259 | 266 |
| C9 | 18348 | 18844 | 2.67 | 2.74 | 11866 | 12082 | 6482 | 6762 | 18037 | 18522 | 311 | 322 |
| Total | 156000 | 156400 |  |  |  |  | 55999 | 56858.09 |  |  |  |  |
| % |  |  |  |  |  |  | 36 | 36 |  |  |  |  |

**Table S3. List of genes mapped to chromosome C7, reference and alternative alleles of SNPs and KASP markers**

| **Gene** | **C7 position** | **Reference allele** | **Alternative allele** | **KASP marker** |
| --- | --- | --- | --- | --- |
| Bo7g106000 | 41028140 | G | C |  |
| Bo7g106000 | 41028170 | A | C |  |
| Bo7g106010 | 41032512 | A | C |  |
| Bo7g106010 | 41032584 | A | C |  |
| Bo7g106010 | 41032605 | A | C |  |
| Bo7g106010 | 41033130 | A | C |  |
| Bo7g106030 | 41039717 | A | C |  |
| Bo7g106060 | 41061117 | A | C |  |
| Bo7g106130 | 41091174 | A | C |  |
| Bo7g106470 | 41313241 | A | C |  |
| Bo7g106470 | 41313306 | A | C |  |
| Bo7g106470 | 41313403 | A | C |  |
| Bo7g106480 | 41327817 | A | C |  |
| Bo7g106490 | 41329788 | A | C |  |
| Bo7g106520 | 41355400 | A | C |  |
| Bo7g106520 | 41357135 | A | C |  |
| Bo7g106590 | 41471221 | A | C |  |
| Bo7g106600 | 41480995 | A | C |  |
| Bo7g106640 | 41493374 | A | C |  |
| Bo7g106640 | 41493522 | A | C |  |
| Bo7g106640 | 41497213 | A | C |  |
| Bo7g106960 | 41695490 | A | C |  |
| Bo7g106960 | 41698263 | A | C |  |
| Bo7g106960 | 41698317 | A | C |  |
| Bo7g107080 | 41811234 | A | C |  |
| Bo7g107100 | 41848312 | A | C |  |
| Bo7g107330 | 41937385 | A | C |  |
| Bo7g107410 | 41993266 | A | C |  |
| Bo7g107450 | 42038831 | A | C |  |
| Bo7g107550 | 42112321 | A | C |  |
| Bo7g107710 | 42200835 | A | C |  |
| Bo7g107770 | 42240162 | A | C |  |
| Bo7g107770 | 42240327 | A | C |  |
| Bo7g107920 | 42328631 | A | C |  |
| Bo7g107940 | 42338844 | A | C |  |
| Bo7g108280 | 42550780 | A | C |  |
| Bo7g108380 | 42600899 | A | C |  |
| Bo7g108380 | 42602252 | A | C |  |
| Bo7g108380 | 42607261 | A | C |  |
| Bo7g108500 | 42706818 | A | C |  |
| Bo7g108630 | 42819372 | A | C |  |
| Bo7g108630 | 42820016 | A | C |  |
| Bo7g108740 | 42863593 | A | C |  |
| Bo7g108740 | 42863938 | A | C |  |
| Bo7g108760 | 42877363 | A | C |  |
| Bo7g108790 | 42888983 | A | C |  |
| Bo7g108820 | 42909051 | A | C |  |
| Bo7g109090 | 43095372 | A | C |  |
| Bo7g109090 | 43095733 | A | C |  |
| Bo7g109100 | 43111958 | A | C |  |
| Bo7g109100 | 43112159 | A | C |  |
| Bo7g109160 | 43147522 | A | C |  |
| Bo7g109160 | 43148100 | A | C |  |
| Bo7g109210 | 43164897 | A | C |  |
| Bo7g109210 | 43165099 | A | C |  |
| Bo7g109210 | 43165532 | A | C |  |
| Bo7g109250 | 43197815 | A | C |  |
| Bo7g109250 | 43198052 | A | C |  |
| Bo7g109290 | 43235907 | A | C |  |
| Bo7g109320 | 43278442 | A | C |  |
| Bo7g109420 | 43315426 | A | C |  |
| Bo7g109420 | 43315459 | A | C |  |
| Bo7g109440 | 43321957 | A | C |  |
| Bo7g109460 | 43332881 | A | C |  |
| Bo7g109460 | 43333156 | A | C |  |
| Bo7g109460 | 43333604 | A | C |  |
| Bo7g109460 | 43334098 | A | C |  |
| Bo7g109490 | 43343769 | A | C |  |
| Bo7g109490 | 43345572 | A | C |  |
| Bo7g109550 | 43374376 | A | C |  |
| Bo7g109690 | 43452795 | A | C |  |
| Bo7g109710 | 43463586 | A | C |  |
| Bo7g109740 | 43474067 | A | C |  |
| Bo7g109760 | 43486490 | A | C |  |
| Bo7g109760 | 43487929 | A | C |  |
| Bo7g109810 | 43503503 | A | C |  |
| Bo7g109810 | 43503521 | A | C |  |
| Bo7g109990 | 43561615 | A | C |  |
| Bo7g109990 | 43562536 | A | C |  |
| Bo7g110050 | 43588113 | A | C |  |
| Bo7g110120 | 43612907 | A | C |  |
| Bo7g110220 | 43651372 | A | C |  |
| Bo7g110240 | 43654511 | A | C |  |
| Bo7g110490 | 43813400 | A | C |  |
| Bo7g110500 | 43819711 | A | C |  |
| Bo7g110530 | 43831739 | A | C |  |
| Bo7g110530 | 43831787 | A | C |  |
| Bo7g106000 | 41029439 | G | C |  |
| Bo7g106010 | 41032569 | G | C |  |
| Bo7g106010 | 41032611 | G | C |  |
| Bo7g106010 | 41032902 | G | C |  |
| Bo7g106010 | 41033262 | G | C |  |
| Bo7g106060 | 41061708 | G | C |  |
| Bo7g106130 | 41091475 | G | C |  |
| Bo7g106130 | 41091741 | G | C |  |
| Bo7g106220 | 41167268 | G | C |  |
| Bo7g106410 | 41275206 | G | C |  |
| Bo7g106440 | 41289472 | G | C |  |
| Bo7g106460 | 41297853 | G | C |  |
| Bo7g106470 | 41313535 | G | C |  |
| Bo7g106480 | 41327894 | G | C |  |
| Bo7g106520 | 41355731 | G | C |  |
| Bo7g106600 | 41481100 | G | C |  |
| Bo7g106620 | 41484863 | G | C |  |
| Bo7g106620 | 41488146 | G | C |  |
| Bo7g106640 | 41493596 | G | C |  |
| Bo7g106640 | 41496602 | G | C |  |
| Bo7g106810 | 41608190 | G | C |  |
| Bo7g106820 | 41622406 | G | C |  |
| Bo7g107080 | 41809232 | G | C |  |
| Bo7g107080 | 41809605 | G | C |  |
| Bo7g107080 | 41810882 | G | C |  |
| Bo7g107100 | 41845065 | G | C |  |
| Bo7g107100 | 41847148 | G | C |  |
| Bo7g107110 | 41849200 | G | C |  |
| Bo7g107110 | 41849278 | G | C |  |
| Bo7g107110 | 41849329 | G | C |  |
| Bo7g107110 | 41849371 | G | C |  |
| Bo7g107110 | 41850097 | G | C |  |
| Bo7g107150 | 41858630 | G | C |  |
| Bo7g107410 | 41994065 | G | C |  |
| Bo7g107520 | 42089303 | G | C |  |
| Bo7g107610 | 42130474 | G | C |  |
| Bo7g107710 | 42200841 | G | C |  |
| Bo7g107710 | 42202412 | G | C |  |
| Bo7g107880 | 42313384 | G | C |  |
| Bo7g107910 | 42322377 | G | C |  |
| Bo7g107930 | 42331911 | G | C |  |
| Bo7g107940 | 42339844 | G | C |  |
| Bo7g108380 | 42602423 | G | C |  |
| Bo7g108570 | 42793532 | G | C |  |
| Bo7g108690 | 42838453 | G | C |  |
| Bo7g108740 | 42863891 | G | C |  |
| Bo7g108820 | 42908967 | G | C |  |
| Bo7g108940 | 43018887 | G | C |  |
| Bo7g109090 | 43094236 | G | C |  |
| Bo7g109090 | 43094432 | G | C |  |
| Bo7g109090 | 43094704 | G | C |  |
| Bo7g109090 | 43094837 | G | C |  |
| Bo7g109090 | 43095303 | G | C |  |
| Bo7g109090 | 43095763 | G | C |  |
| Bo7g109090 | 43095856 | G | C |  |
| Bo7g109100 | 43111895 | G | C |  |
| Bo7g109100 | 43112504 | G | C |  |
| Bo7g109100 | 43112597 | G | C |  |
| Bo7g109160 | 43147163 | G | C |  |
| Bo7g109270 | 43213929 | G | C |  |
| Bo7g109290 | 43234858 | G | C |  |
| Bo7g109390 | 43304960 | G | C |  |
| Bo7g109420 | 43315467 | G | C |  |
| Bo7g109430 | 43317971 | G | C |  |
| Bo7g109460 | 43333083 | G | C |  |
| Bo7g109460 | 43334576 | G | C |  |
| Bo7g109490 | 43344018 | G | C |  |
| Bo7g109500 | 43353797 | G | C |  |
| Bo7g109550 | 43375124 | G | C |  |
| Bo7g109570 | 43378519 | G | C |  |
| Bo7g109610 | 43399571 | G | C |  |
| Bo7g109760 | 43487484 | G | C |  |
| Bo7g109770 | 43489074 | G | C |  |
| Bo7g109900 | 43533155 | G | C |  |
| Bo7g109950 | 43547027 | G | C |  |
| Bo7g109990 | 43563773 | G | C |  |
| Bo7g110240 | 43654578 | G | C |  |
| Bo7g110240 | 43654954 | G | C |  |
| Bo7g110580 | 43857336 | G | C |  |
| Bo7g110630 | 43907270 | G | C |  |
| Bo7g110720 | 43956418 | G | C |  |
| Bo7g106000 | 41028446 | T | C |  |
| Bo7g106000 | 41028614 | T | C |  |
| Bo7g106000 | 41029251 | T | C |  |
| Bo7g106000 | 41029266 | T | C |  |
| Bo7g106010 | 41032425 | T | C |  |
| Bo7g106010 | 41032470 | T | C |  |
| Bo7g106010 | 41032704 | T | C |  |
| Bo7g106010 | 41032710 | T | C |  |
| Bo7g106010 | 41032839 | T | C |  |
| Bo7g106010 | 41033331 | T | C |  |
| Bo7g106020 | 41035756 | T | C |  |
| Bo7g106020 | 41036903 | T | C |  |
| Bo7g106060 | 41060349 | T | C |  |
| Bo7g106060 | 41060418 | T | C |  |
| Bo7g106060 | 41060706 | T | C |  |
| Bo7g106060 | 41061894 | T | C |  |
| Bo7g106130 | 41092685 | T | C |  |
| Bo7g106220 | 41166594 | T | C |  |
| Bo7g106250 | 41179130 | T | C |  |
| Bo7g106320 | 41208905 | T | C |  |
| Bo7g106320 | 41209100 | T | C |  |
| Bo7g106410 | 41275757 | T | C |  |
| Bo7g106410 | 41275769 | T | C |  |
| Bo7g106440 | 41289466 | T | C |  |
| Bo7g106440 | 41289475 | T | C |  |
| Bo7g106450 | 41296509 | T | C |  |
| Bo7g106460 | 41298134 | T | C |  |
| Bo7g106470 | 41313583 | T | C |  |
| Bo7g106480 | 41325967 | T | C |  |
| Bo7g106480 | 41326041 | T | C |  |
| Bo7g106480 | 41326096 | T | C |  |
| Bo7g106480 | 41326144 | T | C |  |
| Bo7g106480 | 41326258 | T | C |  |
| Bo7g106480 | 41326372 | T | C |  |
| Bo7g106480 | 41326711 | T | C |  |
| Bo7g106480 | 41327479 | T | C |  |
| Bo7g106490 | 41329829 | T | C |  |
| Bo7g106520 | 41355049 | T | C |  |
| Bo7g106600 | 41480818 | T | C |  |
| Bo7g106600 | 41481061 | T | C |  |
| Bo7g106620 | 41484534 | T | C |  |
| Bo7g106640 | 41493624 | T | C |  |
| Bo7g106640 | 41493692 | T | C |  |
| Bo7g106640 | 41494295 | T | C |  |
| Bo7g106640 | 41497342 | T | C |  |
| Bo7g106640 | 41498956 | T | C |  |
| Bo7g106640 | 41499204 | T | C |  |
| Bo7g106820 | 41622419 | T | C |  |
| Bo7g106910 | 41679300 | T | C |  |
| Bo7g106910 | 41679305 | T | C |  |
| Bo7g106910 | 41679335 | T | C |  |
| Bo7g106910 | 41679425 | T | C |  |
| Bo7g106910 | 41679489 | T | C |  |
| Bo7g106910 | 41679803 | T | C |  |
| Bo7g106910 | 41680008 | T | C |  |
| Bo7g106960 | 41695568 | T | C |  |
| Bo7g106960 | 41696325 | T | C |  |
| Bo7g106960 | 41697565 | T | C |  |
| Bo7g106960 | 41697779 | T | C |  |
| Bo7g106960 | 41697806 | T | C |  |
| Bo7g107080 | 41809323 | T | C |  |
| Bo7g107080 | 41809902 | T | C |  |
| Bo7g107080 | 41810295 | T | C |  |
| Bo7g107080 | 41810416 | T | C |  |
| Bo7g107100 | 41843099 | T | C |  |
| Bo7g107100 | 41845074 | T | C |  |
| Bo7g107100 | 41848274 | T | C |  |
| Bo7g107110 | 41849599 | T | C |  |
| Bo7g107110 | 41849752 | T | C |  |
| Bo7g107110 | 41849897 | T | C |  |
| Bo7g107110 | 41850187 | T | C |  |
| Bo7g107120 | 41851458 | T | C |  |
| Bo7g107150 | 41858728 | T | C |  |
| Bo7g107150 | 41859024 | T | C |  |
| Bo7g107190 | 41880559 | T | C |  |
| Bo7g107190 | 41880640 | T | C |  |
| Bo7g107190 | 41883110 | T | C |  |
| Bo7g107190 | 41885462 | T | C |  |
| Bo7g107190 | 41885790 | T | C |  |
| Bo7g107190 | 41888408 | T | C |  |
| Bo7g107230 | 41902765 | T | C |  |
| Bo7g107330 | 41936883 | T | C |  |
| Bo7g107330 | 41937409 | T | C |  |
| Bo7g107410 | 41993516 | T | C |  |
| Bo7g107430 | 42026353 | T | C |  |
| Bo7g107450 | 42037253 | T | C |  |
| Bo7g107490 | 42071025 | T | C |  |
| Bo7g107490 | 42071106 | T | C |  |
| Bo7g107490 | 42071836 | T | C |  |
| Bo7g107490 | 42071869 | T | C |  |
| Bo7g107510 | 42086201 | T | C |  |
| Bo7g107510 | 42086232 | T | C |  |
| Bo7g107510 | 42086476 | T | C |  |
| Bo7g107510 | 42086646 | T | C |  |
| Bo7g107550 | 42111371 | T | C |  |
| Bo7g107550 | 42111449 | T | C |  |
| Bo7g107550 | 42111825 | T | C |  |
| Bo7g107550 | 42112388 | T | C |  |
| Bo7g107580 | 42126676 | T | C |  |
| Bo7g107610 | 42130380 | T | C |  |
| Bo7g107610 | 42130471 | T | C |  |
| Bo7g107650 | 42162633 | T | C |  |
| Bo7g107650 | 42163173 | T | C |  |
| Bo7g107710 | 42201183 | T | C |  |
| Bo7g107710 | 42202002 | T | C |  |
| Bo7g107770 | 42240315 | T | C |  |
| Bo7g107810 | 42274653 | T | C |  |
| Bo7g107880 | 42314018 | T | C |  |
| Bo7g107890 | 42317792 | T | C |  |
| Bo7g107890 | 42318334 | T | C |  |
| Bo7g107890 | 42318368 | T | C |  |
| Bo7g107910 | 42321976 | T | C |  |
| Bo7g107920 | 42328469 | T | C |  |
| Bo7g107920 | 42328580 | T | C |  |
| Bo7g107920 | 42328999 | T | C |  |
| Bo7g107930 | 42330137 | T | C |  |
| Bo7g107930 | 42330248 | T | C |  |
| Bo7g107940 | 42339291 | T | C |  |
| Bo7g107990 | 42377084 | T | C |  |
| Bo7g108140 | 42475597 | T | C |  |
| Bo7g108140 | 42475605 | T | C |  |
| Bo7g108200 | 42497102 | T | C |  |
| Bo7g108200 | 42497169 | T | C |  |
| Bo7g108200 | 42498376 | T | C |  |
| Bo7g108280 | 42550852 | T | C |  |
| Bo7g108280 | 42551690 | T | C |  |
| Bo7g108310 | 42570043 | T | C |  |
| Bo7g108350 | 42581612 | T | C | SNP_C7_45 |
| Bo7g108380 | 42598364 | T | C |  |
| Bo7g108380 | 42599582 | T | C |  |
| Bo7g108380 | 42602404 | T | C |  |
| Bo7g108380 | 42602408 | T | C |  |
| Bo7g108380 | 42602510 | T | C |  |
| Bo7g108380 | 42607304 | T | C |  |
| Bo7g108420 | 42637742 | T | C |  |
| Bo7g108500 | 42708039 | T | C |  |
| Bo7g108500 | 42708813 | T | C |  |
| Bo7g108630 | 42820323 | T | C |  |
| Bo7g108690 | 42836187 | T | C |  |
| Bo7g108690 | 42837455 | T | C |  |
| Bo7g108790 | 42886702 | T | C |  |
| Bo7g108790 | 42888686 | T | C |  |
| Bo7g108790 | 42890276 | T | C |  |
| Bo7g108810 | 42893463 | T | C |  |
| Bo7g108810 | 42893848 | T | C |  |
| Bo7g108810 | 42895317 | T | C |  |
| Bo7g108940 | 43018818 | T | C |  |
| Bo7g109040 | 43066792 | T | C |  |
| Bo7g109090 | 43093957 | T | C |  |
| Bo7g109090 | 43094087 | T | C |  |
| Bo7g109090 | 43094159 | T | C |  |
| Bo7g109090 | 43095799 | T | C |  |
| Bo7g109100 | 43111988 | T | C |  |
| Bo7g109100 | 43112171 | T | C |  |
| Bo7g109130 | 43132049 | T | C |  |
| Bo7g109130 | 43132663 | T | C |  |
| Bo7g109130 | 43132672 | T | C |  |
| Bo7g109130 | 43132837 | T | C |  |
| Bo7g109130 | 43132888 | T | C |  |
| Bo7g109130 | 43133086 | T | C |  |
| Bo7g109150 | 43144774 | T | C |  |
| Bo7g109160 | 43145624 | T | C |  |
| Bo7g109160 | 43147129 | T | C |  |
| Bo7g109160 | 43147282 | T | C |  |
| Bo7g109160 | 43147527 | T | C |  |
| Bo7g109160 | 43147553 | T | C |  |
| Bo7g109160 | 43147589 | T | C |  |
| Bo7g109210 | 43165208 | T | C |  |
| Bo7g109250 | 43196743 | T | C |  |
| Bo7g109250 | 43197236 | T | C |  |
| Bo7g109270 | 43213869 | T | C |  |
| Bo7g109270 | 43213875 | T | C |  |
| Bo7g109290 | 43234630 | T | C |  |
| Bo7g109290 | 43234879 | T | C |  |
| Bo7g109290 | 43235865 | T | C |  |
| Bo7g109290 | 43235877 | T | C |  |
| Bo7g109290 | 43236045 | T | C |  |
| Bo7g109290 | 43236398 | T | C |  |
| Bo7g109340 | 43289264 | T | C |  |
| Bo7g109350 | 43293678 | T | C |  |
| Bo7g109390 | 43304301 | T | C |  |
| Bo7g109390 | 43304980 | T | C |  |
| Bo7g109390 | 43304989 | T | C |  |
| Bo7g109390 | 43305261 | T | C |  |
| Bo7g109440 | 43322595 | T | C |  |
| Bo7g109460 | 43332748 | T | C |  |
| Bo7g109460 | 43332825 | T | C |  |
| Bo7g109460 | 43333137 | T | C |  |
| Bo7g109460 | 43333576 | T | C |  |
| Bo7g109460 | 43333616 | T | C |  |
| Bo7g109460 | 43333651 | T | C |  |
| Bo7g109460 | 43333753 | T | C |  |
| Bo7g109490 | 43343562 | T | C |  |
| Bo7g109490 | 43343688 | T | C |  |
| Bo7g109490 | 43343718 | T | C |  |
| Bo7g109490 | 43344207 | T | C |  |
| Bo7g109490 | 43345274 | T | C |  |
| Bo7g109550 | 43373254 | T | C |  |
| Bo7g109550 | 43373854 | T | C |  |
| Bo7g109550 | 43373899 | T | C |  |
| Bo7g109570 | 43378566 | T | C |  |
| Bo7g109570 | 43378671 | T | C |  |
| Bo7g109600 | 43393673 | T | C |  |
| Bo7g109610 | 43396802 | T | C |  |
| Bo7g109610 | 43399303 | T | C |  |
| Bo7g109690 | 43450774 | T | C |  |
| Bo7g109690 | 43451227 | T | C |  |
| Bo7g109690 | 43452043 | T | C |  |
| Bo7g109690 | 43452222 | T | C |  |
| Bo7g109700 | 43459072 | T | C |  |
| Bo7g109700 | 43459339 | T | C |  |
| Bo7g109700 | 43460490 | T | C |  |
| Bo7g109700 | 43461471 | T | C |  |
| Bo7g109710 | 43464109 | T | C |  |
| Bo7g109730 | 43469357 | T | C |  |
| Bo7g109730 | 43469415 | T | C |  |
| Bo7g109750 | 43477776 | T | C |  |
| Bo7g109750 | 43478502 | T | C |  |
| Bo7g109760 | 43485227 | T | C |  |
| Bo7g109760 | 43485233 | T | C |  |
| Bo7g109760 | 43485935 | T | C |  |
| Bo7g109760 | 43486208 | T | C |  |
| Bo7g109760 | 43486587 | T | C |  |
| Bo7g109770 | 43489956 | T | C |  |
| Bo7g109770 | 43490131 | T | C |  |
| Bo7g109800 | 43497993 | T | C |  |
| Bo7g109800 | 43498002 | T | C |  |
| Bo7g109800 | 43498407 | T | C |  |
| Bo7g109800 | 43499263 | T | C |  |
| Bo7g109810 | 43503783 | T | C |  |
| Bo7g109820 | 43505128 | T | C |  |
| Bo7g109820 | 43505669 | T | C |  |
| Bo7g109820 | 43505687 | T | C |  |
| Bo7g109820 | 43505723 | T | C |  |
| Bo7g109820 | 43506929 | T | C |  |
| Bo7g109840 | 43510902 | T | C |  |
| Bo7g109850 | 43514278 | T | C |  |
| Bo7g109900 | 43532221 | T | C |  |
| Bo7g109900 | 43532987 | T | C |  |
| Bo7g109900 | 43533202 | T | C |  |
| Bo7g109920 | 43537742 | T | C |  |
| Bo7g109920 | 43537891 | T | C |  |
| Bo7g110000 | 43568439 | T | C |  |
| Bo7g110050 | 43587914 | T | C |  |
| Bo7g110120 | 43612510 | T | C |  |
| Bo7g110120 | 43612522 | T | C |  |
| Bo7g110190 | 43637734 | T | C |  |
| Bo7g110220 | 43649999 | T | C |  |
| Bo7g110220 | 43650031 | T | C |  |
| Bo7g110260 | 43670005 | T | C |  |
| Bo7g110440 | 43787646 | T | C |  |
| Bo7g110470 | 43803661 | T | C |  |
| Bo7g110500 | 43817239 | T | C |  |
| Bo7g110500 | 43817394 | T | C |  |
| Bo7g110500 | 43818823 | T | C |  |
| Bo7g110500 | 43819219 | T | C |  |
| Bo7g110500 | 43819792 | T | C |  |
| Bo7g110500 | 43820544 | T | C |  |
| Bo7g110500 | 43820841 | T | C |  |
| Bo7g110500 | 43820844 | T | C |  |
| Bo7g110510 | 43822136 | T | C |  |
| Bo7g110530 | 43831784 | T | C |  |
| Bo7g110580 | 43856219 | T | C |  |
| Bo7g110580 | 43857180 | T | C |  |
| Bo7g110630 | 43907448 | T | C |  |
| Bo7g110720 | 43955570 | T | C |  |
| Bo7g110720 | 43956416 | T | C |  |
| Bo7g106440 | 41289487 | A | G |  |
| Bo7g106620 | 41485568 | A | G |  |
| Bo7g106830 | 41626341 | T | G |  |
| Bo7g107520 | 42088485 | A | G |  |
| Bo7g106000 | 41028184 | A | G |  |
| Bo7g106000 | 41029408 | A | G |  |
| Bo7g106010 | 41032599 | A | G |  |
| Bo7g106010 | 41032694 | A | G |  |
| Bo7g106010 | 41032785 | A | G |  |
| Bo7g106010 | 41032788 | A | G |  |
| Bo7g106010 | 41033061 | A | G |  |
| Bo7g106010 | 41033190 | A | G |  |
| Bo7g106010 | 41033274 | A | G |  |
| Bo7g106010 | 41033620 | A | G |  |
| Bo7g106010 | 41033623 | A | G |  |
| Bo7g106030 | 41039938 | A | G |  |
| Bo7g106060 | 41060343 | A | G |  |
| Bo7g106060 | 41060712 | A | G |  |
| Bo7g106060 | 41061126 | A | G |  |
| Bo7g106060 | 41061564 | A | G |  |
| Bo7g106060 | 41061876 | A | G |  |
| Bo7g106130 | 41091208 | A | G |  |
| Bo7g106130 | 41091743 | A | G |  |
| Bo7g106130 | 41093460 | A | G |  |
| Bo7g106130 | 41093661 | A | G |  |
| Bo7g106220 | 41167301 | A | G |  |
| Bo7g106250 | 41179449 | A | G |  |
| Bo7g106250 | 41180136 | A | G |  |
| Bo7g106410 | 41274490 | A | G |  |
| Bo7g106410 | 41274809 | A | G |  |
| Bo7g106410 | 41274887 | A | G |  |
| Bo7g106410 | 41275141 | A | G |  |
| Bo7g106410 | 41275168 | A | G |  |
| Bo7g106450 | 41296190 | A | G |  |
| Bo7g106450 | 41296518 | A | G |  |
| Bo7g106460 | 41298114 | A | G |  |
| Bo7g106460 | 41298670 | A | G |  |
| Bo7g106460 | 41298686 | A | G |  |
| Bo7g106460 | 41298845 | A | G |  |
| Bo7g106460 | 41299383 | A | G |  |
| Bo7g106470 | 41312651 | A | G |  |
| Bo7g106470 | 41314413 | A | G |  |
| Bo7g106480 | 41326054 | A | G |  |
| Bo7g106480 | 41326891 | A | G |  |
| Bo7g106480 | 41327525 | A | G |  |
| Bo7g106600 | 41480797 | A | G |  |
| Bo7g106620 | 41484023 | A | G |  |
| Bo7g106620 | 41484520 | A | G |  |
| Bo7g106620 | 41486348 | A | G |  |
| Bo7g106620 | 41488178 | A | G |  |
| Bo7g106640 | 41493680 | A | G |  |
| Bo7g106640 | 41497409 | A | G |  |
| Bo7g106640 | 41499464 | A | G |  |
| Bo7g106640 | 41499467 | A | G |  |
| Bo7g106640 | 41499488 | A | G |  |
| Bo7g106820 | 41624692 | A | G |  |
| Bo7g106910 | 41679399 | A | G |  |
| Bo7g106910 | 41679566 | A | G |  |
| Bo7g106960 | 41696764 | A | G |  |
| Bo7g106960 | 41696767 | A | G |  |
| Bo7g106960 | 41697507 | A | G |  |
| Bo7g106960 | 41697562 | A | G |  |
| Bo7g107080 | 41810310 | A | G |  |
| Bo7g107080 | 41810608 | A | G |  |
| Bo7g107080 | 41810890 | A | G |  |
| Bo7g107080 | 41811369 | A | G |  |
| Bo7g107100 | 41845947 | A | G |  |
| Bo7g107100 | 41848260 | A | G |  |
| Bo7g107150 | 41858789 | A | G |  |
| Bo7g107150 | 41859002 | A | G |  |
| Bo7g107190 | 41879803 | A | G |  |
| Bo7g107190 | 41881545 | A | G |  |
| Bo7g107190 | 41882656 | A | G |  |
| Bo7g107190 | 41885774 | A | G |  |
| Bo7g107190 | 41886153 | A | G |  |
| Bo7g107190 | 41886353 | A | G |  |
| Bo7g107190 | 41887482 | A | G |  |
| Bo7g107190 | 41888450 | A | G |  |
| Bo7g107190 | 41888559 | A | G |  |
| Bo7g107330 | 41936208 | A | G |  |
| Bo7g107330 | 41937339 | A | G |  |
| Bo7g107410 | 41993855 | A | G |  |
| Bo7g107410 | 41994029 | A | G |  |
| Bo7g107410 | 41994036 | A | G |  |
| Bo7g107430 | 42026388 | A | G |  |
| Bo7g107430 | 42026471 | A | G |  |
| Bo7g107430 | 42026479 | A | G |  |
| Bo7g107450 | 42036952 | A | G |  |
| Bo7g107450 | 42037432 | A | G |  |
| Bo7g107450 | 42039042 | A | G |  |
| Bo7g107450 | 42039201 | A | G |  |
| Bo7g107490 | 42070311 | A | G |  |
| Bo7g107510 | 42086667 | A | G |  |
| Bo7g107510 | 42086913 | A | G |  |
| Bo7g107550 | 42111512 | A | G |  |
| Bo7g107550 | 42111803 | A | G |  |
| Bo7g107550 | 42113755 | A | G |  |
| Bo7g107710 | 42202395 | A | G |  |
| Bo7g107770 | 42240144 | A | G |  |
| Bo7g107770 | 42240465 | A | G |  |
| Bo7g107770 | 42240687 | A | G |  |
| Bo7g107770 | 42241269 | A | G |  |
| Bo7g107810 | 42275619 | A | G |  |
| Bo7g107810 | 42275805 | A | G |  |
| Bo7g107810 | 42276137 | A | G |  |
| Bo7g107810 | 42276152 | A | G |  |
| Bo7g107880 | 42313961 | A | G |  |
| Bo7g107880 | 42314147 | A | G |  |
| Bo7g107890 | 42317578 | A | G |  |
| Bo7g107890 | 42318203 | A | G |  |
| Bo7g107920 | 42328578 | A | G |  |
| Bo7g107940 | 42339849 | A | G |  |
| Bo7g107980 | 42371584 | A | G |  |
| Bo7g108140 | 42474776 | A | G |  |
| Bo7g108200 | 42497989 | A | G |  |
| Bo7g108280 | 42550794 | A | G |  |
| Bo7g108340 | 42579213 | A | G |  |
| Bo7g108350 | 42581775 | A | G |  |
| Bo7g108350 | 42582277 | A | G |  |
| Bo7g108350 | 42582784 | A | G |  |
| Bo7g108380 | 42599449 | A | G |  |
| Bo7g108380 | 42602430 | A | G |  |
| Bo7g108500 | 42706632 | A | G |  |
| Bo7g108500 | 42706677 | A | G |  |
| Bo7g108500 | 42708215 | A | G |  |
| Bo7g108500 | 42708380 | A | G |  |
| Bo7g108500 | 42708603 | A | G |  |
| Bo7g108500 | 42708649 | A | G |  |
| Bo7g108500 | 42708756 | A | G |  |
| Bo7g108630 | 42818563 | A | G |  |
| Bo7g108630 | 42820178 | A | G |  |
| Bo7g108630 | 42820343 | A | G |  |
| Bo7g108690 | 42837433 | A | G |  |
| Bo7g108690 | 42838455 | A | G |  |
| Bo7g108740 | 42863638 | A | G |  |
| Bo7g108760 | 42876895 | A | G |  |
| Bo7g108790 | 42886751 | A | G |  |
| Bo7g108790 | 42886858 | A | G |  |
| Bo7g108790 | 42887412 | A | G |  |
| Bo7g108790 | 42887788 | A | G |  |
| Bo7g108790 | 42887992 | A | G |  |
| Bo7g108790 | 42888839 | A | G |  |
| Bo7g108790 | 42888842 | A | G |  |
| Bo7g108790 | 42889388 | A | G |  |
| Bo7g108790 | 42889603 | A | G |  |
| Bo7g108790 | 42889925 | A | G |  |
| Bo7g108810 | 42893469 | A | G |  |
| Bo7g108820 | 42909246 | A | G |  |
| Bo7g108890 | 42968344 | A | G |  |
| Bo7g108930 | 43015890 | A | G |  |
| Bo7g108940 | 43018415 | A | G |  |
| Bo7g108960 | 43029013 | A | G |  |
| Bo7g109000 | 43044065 | A | G |  |
| Bo7g109080 | 43091931 | A | G |  |
| Bo7g109090 | 43094180 | A | G |  |
| Bo7g109090 | 43094496 | A | G |  |
| Bo7g109090 | 43094774 | A | G |  |
| Bo7g109090 | 43095363 | A | G |  |
| Bo7g109100 | 43112690 | A | G |  |
| Bo7g109130 | 43131694 | A | G |  |
| Bo7g109130 | 43132115 | A | G |  |
| Bo7g109130 | 43132682 | A | G |  |
| Bo7g109130 | 43132693 | A | G |  |
| Bo7g109160 | 43145632 | A | G |  |
| Bo7g109160 | 43146027 | A | G |  |
| Bo7g109160 | 43146976 | A | G |  |
| Bo7g109160 | 43148122 | A | G |  |
| Bo7g109250 | 43197227 | A | G |  |
| Bo7g109250 | 43198270 | A | G |  |
| Bo7g109250 | 43198757 | A | G |  |
| Bo7g109260 | 43208571 | A | G |  |
| Bo7g109270 | 43213532 | A | G |  |
| Bo7g109270 | 43214243 | A | G |  |
| Bo7g109290 | 43234616 | A | G |  |
| Bo7g109290 | 43234847 | A | G |  |
| Bo7g109290 | 43235985 | A | G |  |
| Bo7g109290 | 43237331 | A | G |  |
| Bo7g109320 | 43278498 | A | G |  |
| Bo7g109350 | 43293130 | A | G |  |
| Bo7g109350 | 43293537 | A | G |  |
| Bo7g109350 | 43293681 | A | G |  |
| Bo7g109350 | 43293684 | A | G |  |
| Bo7g109420 | 43314460 | A | G |  |
| Bo7g109430 | 43318296 | A | G |  |
| Bo7g109460 | 43332775 | A | G |  |
| Bo7g109460 | 43333081 | A | G |  |
| Bo7g109460 | 43333579 | A | G |  |
| Bo7g109460 | 43334239 | A | G |  |
| Bo7g109490 | 43343751 | A | G |  |
| Bo7g109490 | 43344300 | A | G |  |
| Bo7g109490 | 43344339 | A | G |  |
| Bo7g109490 | 43344675 | A | G |  |
| Bo7g109490 | 43344913 | A | G |  |
| Bo7g109490 | 43345566 | A | G |  |
| Bo7g109490 | 43345776 | A | G |  |
| Bo7g109500 | 43352478 | A | G |  |
| Bo7g109500 | 43353948 | A | G |  |
| Bo7g109500 | 43353977 | A | G |  |
| Bo7g109540 | 43369611 | A | G |  |
| Bo7g109570 | 43381498 | A | G |  |
| Bo7g109590 | 43385581 | A | G |  |
| Bo7g109590 | 43386466 | A | G |  |
| Bo7g109610 | 43396494 | A | G |  |
| Bo7g109610 | 43399315 | A | G |  |
| Bo7g109610 | 43400682 | A | G |  |
| Bo7g109650 | 43421097 | A | G |  |
| Bo7g109670 | 43433368 | A | G |  |
| Bo7g109670 | 43433880 | A | G |  |
| Bo7g109690 | 43449955 | A | G |  |
| Bo7g109690 | 43452186 | A | G |  |
| Bo7g109690 | 43452781 | A | G |  |
| Bo7g109700 | 43460102 | A | G |  |
| Bo7g109710 | 43464039 | A | G |  |
| Bo7g109710 | 43464617 | A | G |  |
| Bo7g109710 | 43464878 | A | G |  |
| Bo7g109710 | 43465719 | A | G |  |
| Bo7g109710 | 43465907 | A | G |  |
| Bo7g109730 | 43468694 | A | G |  |
| Bo7g109740 | 43474022 | A | G |  |
| Bo7g109740 | 43474759 | A | G |  |
| Bo7g109750 | 43478451 | A | G |  |
| Bo7g109760 | 43485530 | A | G |  |
| Bo7g109760 | 43486601 | A | G |  |
| Bo7g109760 | 43487956 | A | G |  |
| Bo7g109760 | 43487979 | A | G |  |
| Bo7g109770 | 43489106 | A | G |  |
| Bo7g109800 | 43497498 | A | G |  |
| Bo7g109810 | 43502408 | A | G |  |
| Bo7g109810 | 43503468 | A | G |  |
| Bo7g109810 | 43503828 | A | G |  |
| Bo7g109820 | 43506670 | A | G |  |
| Bo7g109850 | 43514395 | A | G |  |
| Bo7g109850 | 43514467 | A | G |  |
| Bo7g109900 | 43533164 | A | G |  |
| Bo7g109920 | 43537974 | A | G |  |
| Bo7g109930 | 43539945 | A | G |  |
| Bo7g109990 | 43563778 | A | G |  |
| Bo7g110120 | 43612909 | A | G |  |
| Bo7g110190 | 43638572 | A | G |  |
| Bo7g110260 | 43669125 | A | G |  |
| Bo7g110450 | 43792582 | A | G |  |
| Bo7g110470 | 43803996 | A | G |  |
| Bo7g110500 | 43817234 | A | G |  |
| Bo7g110500 | 43817812 | A | G |  |
| Bo7g110500 | 43818518 | A | G |  |
| Bo7g110500 | 43818808 | A | G |  |
| Bo7g110500 | 43819173 | A | G |  |
| Bo7g110500 | 43819660 | A | G |  |
| Bo7g110500 | 43820589 | A | G |  |
| Bo7g110580 | 43856340 | A | G |  |
| Bo7g110580 | 43857387 | A | G |  |
| Bo7g110580 | 43858766 | A | G |  |
| Bo7g110630 | 43907765 | A | G |  |
| Bo7g106000 | 41028404 | C | G |  |
| Bo7g106000 | 41028834 | C | G |  |
| Bo7g106000 | 41029460 | C | G |  |
| Bo7g106010 | 41032647 | C | G |  |
| Bo7g106020 | 41037374 | C | G |  |
| Bo7g106060 | 41061651 | C | G |  |
| Bo7g106250 | 41181212 | C | G |  |
| Bo7g106480 | 41326683 | C | G |  |
| Bo7g106480 | 41328351 | C | G |  |
| Bo7g106480 | 41328370 | C | G |  |
| Bo7g106620 | 41488043 | C | G |  |
| Bo7g106640 | 41493416 | C | G |  |
| Bo7g106640 | 41497150 | C | G |  |
| Bo7g106640 | 41498810 | C | G |  |
| Bo7g106930 | 41685259 | C | G |  |
| Bo7g107080 | 41809611 | C | G |  |
| Bo7g107080 | 41812455 | C | G |  |
| Bo7g107110 | 41849557 | C | G |  |
| Bo7g107110 | 41849602 | C | G |  |
| Bo7g107120 | 41851430 | C | G |  |
| Bo7g107190 | 41882863 | C | G |  |
| Bo7g107330 | 41937364 | C | G |  |
| Bo7g107450 | 42039314 | C | G |  |
| Bo7g107490 | 42074054 | C | G |  |
| Bo7g107580 | 42125205 | C | G |  |
| Bo7g107710 | 42201370 | C | G |  |
| Bo7g107770 | 42240615 | C | G |  |
| Bo7g107890 | 42317116 | C | G |  |
| Bo7g107920 | 42328882 | C | G |  |
| Bo7g107920 | 42329101 | C | G |  |
| Bo7g108310 | 42569786 | C | G |  |
| Bo7g108310 | 42569838 | C | G |  |
| Bo7g108310 | 42569848 | C | G |  |
| Bo7g108310 | 42570040 | C | G |  |
| Bo7g108380 | 42598547 | C | G |  |
| Bo7g108380 | 42602256 | C | G |  |
| Bo7g108500 | 42708810 | C | G |  |
| Bo7g108570 | 42793580 | C | G |  |
| Bo7g108630 | 42820379 | C | G |  |
| Bo7g108810 | 42893827 | C | G |  |
| Bo7g108820 | 42906577 | C | G |  |
| Bo7g108940 | 43018131 | C | G |  |
| Bo7g108940 | 43018794 | C | G |  |
| Bo7g108940 | 43018893 | C | G |  |
| Bo7g109100 | 43112623 | C | G |  |
| Bo7g109160 | 43146757 | C | G |  |
| Bo7g109210 | 43164843 | C | G |  |
| Bo7g109250 | 43198057 | C | G |  |
| Bo7g109270 | 43213888 | C | G |  |
| Bo7g109350 | 43293272 | C | G |  |
| Bo7g109420 | 43315229 | C | G |  |
| Bo7g109430 | 43318646 | C | G |  |
| Bo7g109490 | 43343570 | C | G |  |
| Bo7g109490 | 43343757 | C | G |  |
| Bo7g109550 | 43373365 | C | G |  |
| Bo7g109610 | 43396479 | C | G |  |
| Bo7g109610 | 43400688 | C | G |  |
| Bo7g109690 | 43452879 | C | G |  |
| Bo7g109730 | 43468730 | C | G |  |
| Bo7g109740 | 43474025 | C | G |  |
| Bo7g109740 | 43474079 | C | G |  |
| Bo7g109750 | 43478493 | C | G |  |
| Bo7g109760 | 43485563 | C | G |  |
| Bo7g109760 | 43485674 | C | G |  |
| Bo7g109760 | 43486532 | C | G |  |
| Bo7g109800 | 43497951 | C | G |  |
| Bo7g109900 | 43532395 | C | G |  |
| Bo7g109900 | 43533023 | C | G |  |
| Bo7g109900 | 43533230 | C | G |  |
| Bo7g109900 | 43533248 | C | G |  |
| Bo7g109900 | 43533279 | C | G |  |
| Bo7g109990 | 43561058 | C | G |  |
| Bo7g109990 | 43567775 | C | G |  |
| Bo7g110000 | 43568340 | C | G |  |
| Bo7g110190 | 43638476 | C | G |  |
| Bo7g110260 | 43669111 | C | G |  |
| Bo7g110260 | 43670446 | C | G |  |
| Bo7g110260 | 43670513 | C | G |  |
| Bo7g110470 | 43803631 | C | G |  |
| Bo7g110500 | 43817663 | C | G |  |
| Bo7g110500 | 43820496 | C | G |  |
| Bo7g110560 | 43848656 | C | G |  |
| Bo7g110660 | 43915544 | C | G |  |
| Bo7g106000 | 41028242 | T | G |  |
| Bo7g106010 | 41033031 | T | G |  |
| Bo7g106130 | 41091334 | T | G |  |
| Bo7g106130 | 41091403 | T | G |  |
| Bo7g106320 | 41209952 | T | G |  |
| Bo7g106410 | 41274824 | T | G |  |
| Bo7g106440 | 41289686 | T | G |  |
| Bo7g106440 | 41293080 | T | G |  |
| Bo7g106480 | 41326726 | T | G |  |
| Bo7g106480 | 41328284 | T | G |  |
| Bo7g106520 | 41355364 | T | G |  |
| Bo7g106600 | 41481058 | T | G |  |
| Bo7g106620 | 41484865 | T | G |  |
| Bo7g106640 | 41493348 | T | G |  |
| Bo7g106640 | 41493776 | T | G |  |
| Bo7g106640 | 41497374 | T | G |  |
| Bo7g106640 | 41498789 | T | G |  |
| Bo7g106820 | 41624528 | T | G |  |
| Bo7g106830 | 41625628 | T | G |  |
| Bo7g106910 | 41679464 | T | G |  |
| Bo7g107080 | 41810661 | T | G |  |
| Bo7g107080 | 41811237 | T | G |  |
| Bo7g107110 | 41850172 | T | G |  |
| Bo7g107190 | 41881006 | T | G |  |
| Bo7g107190 | 41888510 | T | G |  |
| Bo7g107310 | 41925838 | T | G |  |
| Bo7g107330 | 41936762 | T | G |  |
| Bo7g107430 | 42026441 | T | G |  |
| Bo7g107450 | 42037423 | T | G |  |
| Bo7g107490 | 42071881 | T | G |  |
| Bo7g107550 | 42111938 | T | G |  |
| Bo7g107650 | 42163157 | T | G |  |
| Bo7g107770 | 42240668 | T | G |  |
| Bo7g107770 | 42241096 | T | G |  |
| Bo7g107910 | 42323011 | T | G |  |
| Bo7g107920 | 42329020 | T | G |  |
| Bo7g107930 | 42330930 | T | G |  |
| Bo7g107940 | 42338793 | T | G |  |
| Bo7g108200 | 42497196 | T | G |  |
| Bo7g108200 | 42498064 | T | G |  |
| Bo7g108310 | 42571412 | T | G |  |
| Bo7g108500 | 42708449 | T | G |  |
| Bo7g108500 | 42708698 | T | G |  |
| Bo7g108760 | 42875914 | T | G |  |
| Bo7g108790 | 42887947 | T | G |  |
| Bo7g109000 | 43044068 | T | G |  |
| Bo7g109090 | 43093892 | T | G |  |
| Bo7g109090 | 43094444 | T | G |  |
| Bo7g109090 | 43094606 | T | G |  |
| Bo7g109090 | 43094700 | T | G |  |
| Bo7g109090 | 43095769 | T | G |  |
| Bo7g109100 | 43112099 | T | G |  |
| Bo7g109100 | 43112432 | T | G |  |
| Bo7g109130 | 43131703 | T | G |  |
| Bo7g109130 | 43132849 | T | G |  |
| Bo7g109160 | 43146047 | T | G |  |
| Bo7g109160 | 43147574 | T | G |  |
| Bo7g109160 | 43148168 | T | G |  |
| Bo7g109250 | 43198843 | T | G |  |
| Bo7g109270 | 43214319 | T | G |  |
| Bo7g109290 | 43236056 | T | G |  |
| Bo7g109350 | 43293508 | T | G |  |
| Bo7g109420 | 43315330 | T | G |  |
| Bo7g109460 | 43332867 | T | G |  |
| Bo7g109490 | 43345644 | T | G |  |
| Bo7g109500 | 43353927 | T | G |  |
| Bo7g109570 | 43378665 | T | G |  |
| Bo7g109610 | 43396768 | T | G |  |
| Bo7g109610 | 43400909 | T | G |  |
| Bo7g109690 | 43450799 | T | G |  |
| Bo7g109690 | 43453009 | T | G |  |
| Bo7g109700 | 43461725 | T | G |  |
| Bo7g109700 | 43461847 | T | G |  |
| Bo7g109710 | 43465491 | T | G |  |
| Bo7g109740 | 43474791 | T | G |  |
| Bo7g109750 | 43478820 | T | G |  |
| Bo7g109770 | 43491028 | T | G |  |
| Bo7g109800 | 43497345 | T | G |  |
| Bo7g109800 | 43498361 | T | G |  |
| Bo7g109800 | 43498412 | T | G |  |
| Bo7g109900 | 43532593 | T | G |  |
| Bo7g109930 | 43539942 | T | G |  |
| Bo7g109950 | 43546707 | T | G |  |
| Bo7g109990 | 43562593 | T | G |  |
| Bo7g110120 | 43612310 | T | G |  |
| Bo7g110190 | 43638789 | T | G |  |
| Bo7g110220 | 43650947 | T | G |  |
| Bo7g110500 | 43820030 | T | G |  |
| Bo7g110530 | 43831815 | T | G |  |
| Bo7g110610 | 43872901 | T | G |  |
| Bo7g110740 | 43980463 | T | G |  |
| Bo7g106000 | 41028149 | C | T |  |
| Bo7g107520 | 42089979 | C | T |  |
| Bo7g108150 | 42477626 | A | T |  |
| Bo7g106000 | 41028401 | A | T |  |
| Bo7g106000 | 41029610 | A | T |  |
| Bo7g106010 | 41033160 | A | T |  |
| Bo7g106010 | 41033304 | A | T |  |
| Bo7g106130 | 41092200 | A | T |  |
| Bo7g106250 | 41178956 | A | T |  |
| Bo7g106250 | 41179527 | A | T |  |
| Bo7g106250 | 41181591 | A | T |  |
| Bo7g106320 | 41209832 | A | T |  |
| Bo7g106410 | 41274387 | A | T |  |
| Bo7g106410 | 41274568 | A | T |  |
| Bo7g106450 | 41296071 | A | T |  |
| Bo7g106460 | 41298041 | A | T |  |
| Bo7g106460 | 41298791 | A | T |  |
| Bo7g106480 | 41326681 | A | T |  |
| Bo7g106480 | 41327883 | A | T |  |
| Bo7g106640 | 41493975 | A | T |  |
| Bo7g106640 | 41497332 | A | T |  |
| Bo7g106820 | 41624548 | A | T |  |
| Bo7g106910 | 41679779 | A | T |  |
| Bo7g106960 | 41697491 | A | T |  |
| Bo7g106960 | 41698080 | A | T |  |
| Bo7g107080 | 41809409 | A | T |  |
| Bo7g107080 | 41809416 | A | T |  |
| Bo7g107080 | 41809749 | A | T |  |
| Bo7g107080 | 41811249 | A | T |  |
| Bo7g107150 | 41858726 | A | T |  |
| Bo7g107190 | 41881265 | A | T |  |
| Bo7g107190 | 41885565 | A | T |  |
| Bo7g107190 | 41886614 | A | T |  |
| Bo7g107330 | 41937361 | A | T |  |
| Bo7g107450 | 42036911 | A | T |  |
| Bo7g107490 | 42074016 | A | T |  |
| Bo7g107550 | 42111561 | A | T |  |
| Bo7g107580 | 42126164 | A | T |  |
| Bo7g107650 | 42163177 | A | T |  |
| Bo7g107770 | 42240000 | A | T |  |
| Bo7g107890 | 42317575 | A | T |  |
| Bo7g107920 | 42329008 | A | T |  |
| Bo7g108280 | 42550145 | A | T |  |
| Bo7g108280 | 42550840 | A | T |  |
| Bo7g108380 | 42598334 | A | T |  |
| Bo7g108380 | 42598454 | A | T |  |
| Bo7g108380 | 42602508 | A | T |  |
| Bo7g108630 | 42818373 | A | T |  |
| Bo7g108630 | 42819348 | A | T |  |
| Bo7g108630 | 42820230 | A | T |  |
| Bo7g108810 | 42895608 | A | T |  |
| Bo7g109090 | 43094132 | A | T |  |
| Bo7g109100 | 43112066 | A | T |  |
| Bo7g109100 | 43112402 | A | T |  |
| Bo7g109130 | 43132107 | A | T |  |
| Bo7g109210 | 43165553 | A | T |  |
| Bo7g109210 | 43165586 | A | T |  |
| Bo7g109270 | 43213619 | A | T |  |
| Bo7g109270 | 43213811 | A | T |  |
| Bo7g109290 | 43234972 | A | T |  |
| Bo7g109350 | 43292895 | A | T |  |
| Bo7g109350 | 43293846 | A | T |  |
| Bo7g109420 | 43314426 | A | T |  |
| Bo7g109420 | 43315281 | A | T |  |
| Bo7g109420 | 43315348 | A | T |  |
| Bo7g109460 | 43333159 | A | T |  |
| Bo7g109460 | 43334275 | A | T |  |
| Bo7g109490 | 43343628 | A | T |  |
| Bo7g109490 | 43344952 | A | T |  |
| Bo7g109500 | 43352045 | A | T |  |
| Bo7g109500 | 43353893 | A | T |  |
| Bo7g109530 | 43366951 | A | T |  |
| Bo7g109650 | 43421916 | A | T |  |
| Bo7g109690 | 43452082 | A | T |  |
| Bo7g109700 | 43459045 | A | T |  |
| Bo7g109700 | 43461470 | A | T |  |
| Bo7g109700 | 43461995 | A | T |  |
| Bo7g109750 | 43478771 | A | T |  |
| Bo7g109760 | 43487431 | A | T |  |
| Bo7g109800 | 43497999 | A | T |  |
| Bo7g109810 | 43500678 | A | T |  |
| Bo7g109820 | 43506637 | A | T |  |
| Bo7g109990 | 43561107 | A | T |  |
| Bo7g109990 | 43561115 | A | T |  |
| Bo7g109990 | 43562511 | A | T |  |
| Bo7g109990 | 43566341 | A | T |  |
| Bo7g110240 | 43654945 | A | T |  |
| Bo7g110500 | 43817874 | A | T |  |
| Bo7g110510 | 43822223 | A | T |  |
| Bo7g106000 | 41028210 | C | T |  |
| Bo7g106000 | 41028849 | C | T |  |
| Bo7g106000 | 41029210 | C | T |  |
| Bo7g106000 | 41029669 | C | T |  |
| Bo7g106010 | 41033022 | C | T |  |
| Bo7g106010 | 41033028 | C | T |  |
| Bo7g106030 | 41039918 | C | T |  |
| Bo7g106060 | 41061057 | C | T |  |
| Bo7g106130 | 41091157 | C | T |  |
| Bo7g106130 | 41092413 | C | T |  |
| Bo7g106220 | 41165992 | C | T |  |
| Bo7g106220 | 41166127 | C | T |  |
| Bo7g106220 | 41166639 | C | T |  |
| Bo7g106250 | 41179124 | C | T |  |
| Bo7g106250 | 41180082 | C | T |  |
| Bo7g106410 | 41274851 | C | T |  |
| Bo7g106410 | 41275227 | C | T |  |
| Bo7g106410 | 41275807 | C | T |  |
| Bo7g106460 | 41297768 | C | T |  |
| Bo7g106470 | 41312627 | C | T |  |
| Bo7g106470 | 41314604 | C | T |  |
| Bo7g106470 | 41314645 | C | T |  |
| Bo7g106480 | 41326423 | C | T |  |
| Bo7g106480 | 41327146 | C | T |  |
| Bo7g106480 | 41327825 | C | T |  |
| Bo7g106480 | 41328345 | C | T |  |
| Bo7g106480 | 41328399 | C | T |  |
| Bo7g106490 | 41330090 | C | T |  |
| Bo7g106520 | 41355346 | C | T |  |
| Bo7g106600 | 41480050 | C | T |  |
| Bo7g106600 | 41480062 | C | T |  |
| Bo7g106600 | 41480329 | C | T |  |
| Bo7g106600 | 41480338 | C | T |  |
| Bo7g106620 | 41485031 | C | T |  |
| Bo7g106620 | 41487971 | C | T |  |
| Bo7g106630 | 41489085 | C | T |  |
| Bo7g106640 | 41493770 | C | T |  |
| Bo7g106640 | 41493773 | C | T |  |
| Bo7g106640 | 41494187 | C | T |  |
| Bo7g106640 | 41498057 | C | T |  |
| Bo7g106910 | 41679542 | C | T |  |
| Bo7g106910 | 41679869 | C | T |  |
| Bo7g106960 | 41695595 | C | T |  |
| Bo7g106960 | 41696907 | C | T |  |
| Bo7g106960 | 41696925 | C | T |  |
| Bo7g106960 | 41697124 | C | T |  |
| Bo7g106960 | 41697257 | C | T |  |
| Bo7g106960 | 41697505 | C | T |  |
| Bo7g106960 | 41697583 | C | T |  |
| Bo7g106960 | 41698004 | C | T |  |
| Bo7g106960 | 41698275 | C | T |  |
| Bo7g107080 | 41809431 | C | T |  |
| Bo7g107080 | 41809800 | C | T |  |
| Bo7g107080 | 41810394 | C | T |  |
| Bo7g107080 | 41810403 | C | T |  |
| Bo7g107080 | 41810464 | C | T |  |
| Bo7g107080 | 41810868 | C | T |  |
| Bo7g107080 | 41812548 | C | T |  |
| Bo7g107110 | 41849110 | C | T |  |
| Bo7g107110 | 41849386 | C | T |  |
| Bo7g107110 | 41849536 | C | T |  |
| Bo7g107110 | 41849866 | C | T |  |
| Bo7g107110 | 41850232 | C | T |  |
| Bo7g107190 | 41880778 | C | T |  |
| Bo7g107190 | 41885568 | C | T |  |
| Bo7g107190 | 41885772 | C | T |  |
| Bo7g107190 | 41885874 | C | T |  |
| Bo7g107190 | 41887494 | C | T |  |
| Bo7g107310 | 41925874 | C | T |  |
| Bo7g107330 | 41936891 | C | T |  |
| Bo7g107330 | 41936900 | C | T |  |
| Bo7g107450 | 42036940 | C | T |  |
| Bo7g107450 | 42038678 | C | T |  |
| Bo7g107490 | 42071068 | C | T |  |
| Bo7g107490 | 42071094 | C | T |  |
| Bo7g107490 | 42071824 | C | T |  |
| Bo7g107490 | 42072262 | C | T |  |
| Bo7g107510 | 42086262 | C | T |  |
| Bo7g107550 | 42111470 | C | T |  |
| Bo7g107550 | 42112088 | C | T |  |
| Bo7g107550 | 42112563 | C | T |  |
| Bo7g107610 | 42131633 | C | T |  |
| Bo7g107650 | 42163305 | C | T |  |
| Bo7g107770 | 42240235 | C | T |  |
| Bo7g107770 | 42240648 | C | T |  |
| Bo7g107880 | 42313434 | C | T |  |
| Bo7g107890 | 42317533 | C | T |  |
| Bo7g107890 | 42317735 | C | T |  |
| Bo7g107910 | 42321963 | C | T |  |
| Bo7g107910 | 42322372 | C | T |  |
| Bo7g107920 | 42328857 | C | T |  |
| Bo7g107920 | 42329032 | C | T |  |
| Bo7g107930 | 42330388 | C | T |  |
| Bo7g107930 | 42331809 | C | T |  |
| Bo7g107930 | 42331815 | C | T |  |
| Bo7g107930 | 42331851 | C | T |  |
| Bo7g107930 | 42332178 | C | T |  |
| Bo7g108200 | 42497850 | C | T |  |
| Bo7g108310 | 42569762 | C | T |  |
| Bo7g108310 | 42570762 | C | T |  |
| Bo7g108310 | 42570774 | C | T |  |
| Bo7g108310 | 42571212 | C | T |  |
| Bo7g108350 | 42581810 | C | T |  |
| Bo7g108350 | 42583000 | C | T |  |
| Bo7g108380 | 42598589 | C | T |  |
| Bo7g108380 | 42599492 | C | T |  |
| Bo7g108380 | 42602519 | C | T |  |
| Bo7g108500 | 42706058 | C | T |  |
| Bo7g108500 | 42707038 | C | T |  |
| Bo7g108630 | 42819390 | C | T |  |
| Bo7g108630 | 42820205 | C | T |  |
| Bo7g108630 | 42820345 | C | T |  |
| Bo7g108690 | 42838449 | C | T |  |
| Bo7g108740 | 42863845 | C | T |  |
| Bo7g108790 | 42886754 | C | T |  |
| Bo7g108790 | 42887446 | C | T | SNP_C7_43 |
| Bo7g108790 | 42887755 | C | T |  |
| Bo7g108790 | 42888980 | C | T |  |
| Bo7g108790 | 42889307 | C | T | SNP_C7_34 |
| Bo7g108790 | 42889397 | C | T |  |
| Bo7g108810 | 42894831 | C | T |  |
| Bo7g108810 | 42894873 | C | T |  |
| Bo7g108820 | 42906589 | C | T |  |
| Bo7g108820 | 42908702 | C | T |  |
| Bo7g108820 | 42908883 | C | T |  |
| Bo7g108890 | 42968310 | C | T |  |
| Bo7g108890 | 42968463 | C | T |  |
| Bo7g108930 | 43015129 | C | T |  |
| Bo7g108940 | 43017949 | C | T |  |
| Bo7g108940 | 43018635 | C | T |  |
| Bo7g108940 | 43020238 | C | T |  |
| Bo7g108960 | 43028977 | C | T |  |
| Bo7g109090 | 43094156 | C | T |  |
| Bo7g109090 | 43094790 | C | T |  |
| Bo7g109120 | 43128972 | C | T |  |
| Bo7g109130 | 43131613 | C | T |  |
| Bo7g109130 | 43132283 | C | T |  |
| Bo7g109160 | 43146038 | C | T |  |
| Bo7g109160 | 43146404 | C | T |  |
| Bo7g109160 | 43147138 | C | T |  |
| Bo7g109160 | 43147327 | C | T |  |
| Bo7g109160 | 43147922 | C | T |  |
| Bo7g109210 | 43165535 | C | T |  |
| Bo7g109250 | 43196818 | C | T |  |
| Bo7g109250 | 43197386 | C | T |  |
| Bo7g109250 | 43198060 | C | T |  |
| Bo7g109270 | 43213526 | C | T |  |
| Bo7g109270 | 43213614 | C | T |  |
| Bo7g109270 | 43214005 | C | T |  |
| Bo7g109270 | 43214115 | C | T |  |
| Bo7g109270 | 43214233 | C | T |  |
| Bo7g109270 | 43214314 | C | T |  |
| Bo7g109270 | 43214323 | C | T |  |
| Bo7g109290 | 43235023 | C | T |  |
| Bo7g109290 | 43235934 | C | T |  |
| Bo7g109340 | 43289165 | C | T |  |
| Bo7g109350 | 43293750 | C | T |  |
| Bo7g109350 | 43293825 | C | T |  |
| Bo7g109390 | 43304340 | C | T |  |
| Bo7g109420 | 43315273 | C | T |  |
| Bo7g109430 | 43318491 | C | T |  |
| Bo7g109430 | 43318550 | C | T |  |
| Bo7g109440 | 43320393 | C | T |  |
| Bo7g109460 | 43332999 | C | T |  |
| Bo7g109460 | 43333816 | C | T |  |
| Bo7g109490 | 43343646 | C | T |  |
| Bo7g109490 | 43344165 | C | T |  |
| Bo7g109490 | 43344264 | C | T |  |
| Bo7g109490 | 43344919 | C | T |  |
| Bo7g109490 | 43345205 | C | T |  |
| Bo7g109490 | 43345539 | C | T |  |
| Bo7g109490 | 43345623 | C | T |  |
| Bo7g109500 | 43352051 | C | T |  |
| Bo7g109500 | 43352409 | C | T |  |
| Bo7g109500 | 43352472 | C | T |  |
| Bo7g109500 | 43354127 | C | T |  |
| Bo7g109500 | 43354142 | C | T |  |
| Bo7g109550 | 43373263 | C | T |  |
| Bo7g109550 | 43375144 | C | T |  |
| Bo7g109600 | 43394014 | C | T |  |
| Bo7g109670 | 43433946 | C | T |  |
| Bo7g109690 | 43450753 | C | T |  |
| Bo7g109690 | 43451567 | C | T |  |
| Bo7g109690 | 43452906 | C | T |  |
| Bo7g109690 | 43453096 | C | T |  |
| Bo7g109700 | 43461504 | C | T |  |
| Bo7g109710 | 43463634 | C | T |  |
| Bo7g109710 | 43464030 | C | T |  |
| Bo7g109710 | 43464872 | C | T |  |
| Bo7g109710 | 43465280 | C | T |  |
| Bo7g109710 | 43465506 | C | T |  |
| Bo7g109730 | 43468109 | C | T |  |
| Bo7g109740 | 43474040 | C | T |  |
| Bo7g109740 | 43474055 | C | T |  |
| Bo7g109740 | 43474369 | C | T |  |
| Bo7g109750 | 43477866 | C | T |  |
| Bo7g109760 | 43485629 | C | T |  |
| Bo7g109760 | 43486802 | C | T |  |
| Bo7g109760 | 43487622 | C | T |  |
| Bo7g109770 | 43488885 | C | T |  |
| Bo7g109770 | 43489950 | C | T |  |
| Bo7g109800 | 43497144 | C | T |  |
| Bo7g109800 | 43497351 | C | T |  |
| Bo7g109800 | 43498026 | C | T |  |
| Bo7g109800 | 43498397 | C | T |  |
| Bo7g109800 | 43499276 | C | T |  |
| Bo7g109810 | 43502806 | C | T |  |
| Bo7g109810 | 43503465 | C | T |  |
| Bo7g109810 | 43504178 | C | T |  |
| Bo7g109820 | 43506709 | C | T |  |
| Bo7g109820 | 43506843 | C | T |  |
| Bo7g109820 | 43506948 | C | T |  |
| Bo7g109850 | 43513891 | C | T |  |
| Bo7g109850 | 43514212 | C | T |  |
| Bo7g109900 | 43532173 | C | T |  |
| Bo7g109900 | 43532617 | C | T |  |
| Bo7g109900 | 43532711 | C | T |  |
| Bo7g109920 | 43538022 | C | T |  |
| Bo7g109990 | 43565745 | C | T |  |
| Bo7g109990 | 43566570 | C | T |  |
| Bo7g109990 | 43566578 | C | T |  |
| Bo7g109990 | 43566896 | C | T |  |
| Bo7g110050 | 43587728 | C | T |  |
| Bo7g110050 | 43588073 | C | T |  |
| Bo7g110120 | 43612353 | C | T |  |
| Bo7g110120 | 43612393 | C | T |  |
| Bo7g110190 | 43638175 | C | T |  |
| Bo7g110190 | 43638192 | C | T |  |
| Bo7g110190 | 43638760 | C | T |  |
| Bo7g110220 | 43651377 | C | T |  |
| Bo7g110260 | 43670065 | C | T |  |
| Bo7g110260 | 43670068 | C | T |  |
| Bo7g110450 | 43792519 | C | T |  |
| Bo7g110450 | 43793263 | C | T |  |
| Bo7g110470 | 43804637 | C | T |  |
| Bo7g110500 | 43817280 | C | T |  |
| Bo7g110500 | 43817640 | C | T |  |
| Bo7g110500 | 43817880 | C | T |  |
| Bo7g110500 | 43818299 | C | T |  |
| Bo7g110500 | 43819881 | C | T |  |
| Bo7g110510 | 43822199 | C | T |  |
| Bo7g110530 | 43831733 | C | T |  |
| Bo7g110560 | 43848792 | C | T |  |
| Bo7g110580 | 43856156 | C | T |  |
| Bo7g110580 | 43856183 | C | T |  |
| Bo7g110580 | 43856453 | C | T |  |
| Bo7g110580 | 43857567 | C | T |  |
| Bo7g110580 | 43858799 | C | T |  |
| Bo7g110630 | 43906495 | C | T |  |
| Bo7g106130 | 41091152 | G | T |  |
| Bo7g106130 | 41091748 | G | T |  |
| Bo7g106220 | 41166434 | G | T |  |
| Bo7g106470 | 41313589 | G | T |  |
| Bo7g106480 | 41325943 | G | T |  |
| Bo7g106480 | 41327095 | G | T |  |
| Bo7g106480 | 41327122 | G | T |  |
| Bo7g106490 | 41329742 | G | T |  |
| Bo7g106520 | 41355052 | G | T |  |
| Bo7g106620 | 41487943 | G | T |  |
| Bo7g106620 | 41488112 | G | T |  |
| Bo7g106620 | 41488229 | G | T |  |
| Bo7g106630 | 41489235 | G | T |  |
| Bo7g106640 | 41494873 | G | T |  |
| Bo7g106960 | 41695590 | G | T |  |
| Bo7g106960 | 41696937 | G | T |  |
| Bo7g106960 | 41697475 | G | T |  |
| Bo7g106960 | 41698260 | G | T |  |
| Bo7g107080 | 41810098 | G | T |  |
| Bo7g107190 | 41886596 | G | T |  |
| Bo7g107330 | 41937231 | G | T |  |
| Bo7g107450 | 42037246 | G | T |  |
| Bo7g107550 | 42111738 | G | T |  |
| Bo7g107550 | 42112419 | G | T |  |
| Bo7g107550 | 42113908 | G | T |  |
| Bo7g107650 | 42162656 | G | T |  |
| Bo7g107810 | 42276154 | G | T |  |
| Bo7g108140 | 42475304 | G | T |  |
| Bo7g108420 | 42641140 | G | T |  |
| Bo7g108630 | 42818439 | G | T |  |
| Bo7g108740 | 42863903 | G | T |  |
| Bo7g108760 | 42875964 | G | T |  |
| Bo7g108790 | 42886855 | G | T |  |
| Bo7g108790 | 42887406 | G | T |  |
| Bo7g108810 | 42894007 | G | T |  |
| Bo7g108810 | 42895014 | G | T |  |
| Bo7g108820 | 42909171 | G | T |  |
| Bo7g109090 | 43094012 | G | T |  |
| Bo7g109090 | 43094678 | G | T |  |
| Bo7g109100 | 43111835 | G | T |  |
| Bo7g109120 | 43128999 | G | T |  |
| Bo7g109130 | 43133038 | G | T |  |
| Bo7g109160 | 43148136 | G | T |  |
| Bo7g109210 | 43165072 | G | T |  |
| Bo7g109210 | 43165075 | G | T |  |
| Bo7g109350 | 43293585 | G | T |  |
| Bo7g109460 | 43333863 | G | T |  |
| Bo7g109490 | 43345277 | G | T |  |
| Bo7g109500 | 43354115 | G | T |  |
| Bo7g109550 | 43374312 | G | T |  |
| Bo7g109570 | 43378658 | G | T |  |
| Bo7g109610 | 43399859 | G | T |  |
| Bo7g109650 | 43420603 | G | T |  |
| Bo7g109670 | 43433280 | G | T |  |
| Bo7g109700 | 43460214 | G | T |  |
| Bo7g109700 | 43460943 | G | T |  |
| Bo7g109700 | 43461226 | G | T |  |
| Bo7g109700 | 43461394 | G | T |  |
| Bo7g109710 | 43465301 | G | T |  |
| Bo7g109750 | 43478430 | G | T |  |
| Bo7g109760 | 43486559 | G | T |  |
| Bo7g109800 | 43497492 | G | T |  |
| Bo7g109810 | 43503484 | G | T |  |
| Bo7g109810 | 43503785 | G | T |  |
| Bo7g109920 | 43537697 | G | T |  |
| Bo7g110220 | 43650065 | G | T |  |
| Bo7g110220 | 43651434 | G | T |  |
| Bo7g110240 | 43654503 | G | T |  |
| Bo7g110500 | 43817997 | G | T |  |
| Bo7g110500 | 43819093 | G | T |  |
| Bo7g110560 | 43849581 | G | T |  |
| Bo7g110610 | 43872688 | G | T |  |
| Bo7g106020 | 41036720 | C | A |  |
| Bo7g106620 | 41486411 | G | A |  |
| Bo7g107470 | 42042893 | G | A |  |
| Bo7g106010 | 41032758 | C | A |  |
| Bo7g106060 | 41061114 | C | A |  |
| Bo7g106060 | 41061270 | C | A |  |
| Bo7g106060 | 41061429 | C | A |  |
| Bo7g106250 | 41179100 | C | A |  |
| Bo7g106320 | 41209991 | C | A |  |
| Bo7g106410 | 41275108 | C | A |  |
| Bo7g106410 | 41275764 | C | A |  |
| Bo7g106450 | 41296539 | C | A |  |
| Bo7g106460 | 41298063 | C | A |  |
| Bo7g106460 | 41298111 | C | A |  |
| Bo7g106460 | 41298683 | C | A |  |
| Bo7g106520 | 41357371 | C | A |  |
| Bo7g106600 | 41480301 | C | A |  |
| Bo7g106600 | 41481052 | C | A |  |
| Bo7g106620 | 41484018 | C | A |  |
| Bo7g106820 | 41622594 | C | A |  |
| Bo7g106820 | 41623391 | C | A |  |
| Bo7g106830 | 41625562 | C | A |  |
| Bo7g106910 | 41680344 | C | A |  |
| Bo7g106960 | 41697320 | C | A |  |
| Bo7g107080 | 41809551 | C | A |  |
| Bo7g107100 | 41848279 | C | A |  |
| Bo7g107190 | 41888621 | C | A |  |
| Bo7g107330 | 41937018 | C | A |  |
| Bo7g107450 | 42038833 | C | A |  |
| Bo7g107450 | 42039318 | C | A |  |
| Bo7g107490 | 42071062 | C | A |  |
| Bo7g107550 | 42113012 | C | A |  |
| Bo7g107550 | 42113812 | C | A |  |
| Bo7g107710 | 42201528 | C | A |  |
| Bo7g107770 | 42241430 | C | A |  |
| Bo7g107890 | 42317112 | C | A |  |
| Bo7g107920 | 42328463 | C | A |  |
| Bo7g107920 | 42329232 | C | A |  |
| Bo7g108280 | 42551014 | C | A |  |
| Bo7g108350 | 42582805 | C | A |  |
| Bo7g108380 | 42599430 | C | A |  |
| Bo7g108380 | 42600950 | C | A |  |
| Bo7g108380 | 42602241 | C | A |  |
| Bo7g108570 | 42793529 | C | A |  |
| Bo7g108630 | 42820282 | C | A |  |
| Bo7g108690 | 42836151 | C | A |  |
| Bo7g108790 | 42889168 | C | A |  |
| Bo7g108820 | 42908878 | C | A |  |
| Bo7g108960 | 43028982 | C | A |  |
| Bo7g109090 | 43094738 | C | A | SNP_C7_56 |
| Bo7g109100 | 43112589 | C | A |  |
| Bo7g109120 | 43129435 | C | A |  |
| Bo7g109130 | 43132846 | C | A |  |
| Bo7g109260 | 43208416 | C | A |  |
| Bo7g109270 | 43214288 | C | A |  |
| Bo7g109290 | 43235950 | C | A |  |
| Bo7g109440 | 43320428 | C | A |  |
| Bo7g109460 | 43333572 | C | A |  |
| Bo7g109490 | 43345638 | C | A |  |
| Bo7g109550 | 43373904 | C | A |  |
| Bo7g109610 | 43396856 | C | A |  |
| Bo7g109610 | 43397544 | C | A |  |
| Bo7g109700 | 43459552 | C | A |  |
| Bo7g109700 | 43461737 | C | A |  |
| Bo7g109700 | 43462059 | C | A |  |
| Bo7g109710 | 43464785 | C | A |  |
| Bo7g109710 | 43465810 | C | A |  |
| Bo7g109760 | 43485083 | C | A |  |
| Bo7g109760 | 43487385 | C | A |  |
| Bo7g109760 | 43487958 | C | A |  |
| Bo7g109770 | 43489062 | C | A |  |
| Bo7g109920 | 43537733 | C | A |  |
| Bo7g109990 | 43566314 | C | A |  |
| Bo7g110190 | 43638575 | C | A |  |
| Bo7g110190 | 43638797 | C | A |  |
| Bo7g110220 | 43651389 | C | A |  |
| Bo7g110500 | 43817445 | C | A |  |
| Bo7g110510 | 43822130 | C | A |  |
| Bo7g110580 | 43856280 | C | A |  |
| Bo7g110630 | 43906159 | C | A |  |
| Bo7g110630 | 43906816 | C | A |  |
| Bo7g110630 | 43907566 | C | A |  |
| Bo7g106000 | 41028906 | G | A |  |
| Bo7g106010 | 41032488 | G | A |  |
| Bo7g106010 | 41032506 | G | A |  |
| Bo7g106060 | 41061090 | G | A |  |
| Bo7g106060 | 41061120 | G | A |  |
| Bo7g106060 | 41061294 | G | A |  |
| Bo7g106060 | 41061303 | G | A |  |
| Bo7g106060 | 41061432 | G | A |  |
| Bo7g106060 | 41061597 | G | A |  |
| Bo7g106060 | 41061600 | G | A |  |
| Bo7g106130 | 41091241 | G | A |  |
| Bo7g106130 | 41091468 | G | A |  |
| Bo7g106130 | 41093493 | G | A |  |
| Bo7g106220 | 41166025 | G | A |  |
| Bo7g106250 | 41179356 | G | A |  |
| Bo7g106250 | 41179419 | G | A |  |
| Bo7g106250 | 41181208 | G | A |  |
| Bo7g106250 | 41181221 | G | A |  |
| Bo7g106450 | 41296035 | G | A |  |
| Bo7g106460 | 41298177 | G | A |  |
| Bo7g106460 | 41299608 | G | A |  |
| Bo7g106470 | 41312801 | G | A |  |
| Bo7g106470 | 41313160 | G | A |  |
| Bo7g106470 | 41313292 | G | A |  |
| Bo7g106480 | 41326324 | G | A |  |
| Bo7g106480 | 41326327 | G | A |  |
| Bo7g106480 | 41327059 | G | A |  |
| Bo7g106480 | 41327143 | G | A |  |
| Bo7g106480 | 41327734 | G | A |  |
| Bo7g106480 | 41327860 | G | A |  |
| Bo7g106480 | 41328183 | G | A |  |
| Bo7g106490 | 41329760 | G | A |  |
| Bo7g106510 | 41351946 | G | A |  |
| Bo7g106520 | 41355349 | G | A |  |
| Bo7g106520 | 41356543 | G | A |  |
| Bo7g106520 | 41357210 | G | A |  |
| Bo7g106590 | 41471241 | G | A |  |
| Bo7g106600 | 41480102 | G | A |  |
| Bo7g106600 | 41480770 | G | A |  |
| Bo7g106600 | 41481087 | G | A |  |
| Bo7g106640 | 41493689 | G | A |  |
| Bo7g106640 | 41496311 | G | A |  |
| Bo7g106640 | 41496635 | G | A |  |
| Bo7g106640 | 41497384 | G | A |  |
| Bo7g106640 | 41497787 | G | A |  |
| Bo7g106820 | 41622609 | G | A |  |
| Bo7g106830 | 41625559 | G | A |  |
| Bo7g106830 | 41625640 | G | A |  |
| Bo7g106910 | 41680044 | G | A |  |
| Bo7g106910 | 41680051 | G | A |  |
| Bo7g106910 | 41680088 | G | A |  |
| Bo7g106910 | 41680099 | G | A |  |
| Bo7g106960 | 41695517 | G | A |  |
| Bo7g106960 | 41696910 | G | A |  |
| Bo7g106960 | 41697092 | G | A |  |
| Bo7g106960 | 41697729 | G | A |  |
| Bo7g107100 | 41844183 | G | A |  |
| Bo7g107100 | 41844624 | G | A |  |
| Bo7g107110 | 41849671 | G | A |  |
| Bo7g107190 | 41880761 | G | A |  |
| Bo7g107190 | 41886060 | G | A |  |
| Bo7g107190 | 41888315 | G | A |  |
| Bo7g107210 | 41892383 | G | A |  |
| Bo7g107330 | 41936820 | G | A |  |
| Bo7g107410 | 41994145 | G | A |  |
| Bo7g107450 | 42037249 | G | A |  |
| Bo7g107490 | 42073194 | G | A |  |
| Bo7g107490 | 42073197 | G | A |  |
| Bo7g107490 | 42074014 | G | A |  |
| Bo7g107490 | 42074559 | G | A |  |
| Bo7g107510 | 42086184 | G | A |  |
| Bo7g107550 | 42111835 | G | A | SNP_C7_20 |
| Bo7g107550 | 42112690 | G | A |  |
| Bo7g107550 | 42112865 | G | A |  |
| Bo7g107550 | 42112994 | G | A |  |
| Bo7g107550 | 42113220 | G | A |  |
| Bo7g107550 | 42113725 | G | A |  |
| Bo7g107550 | 42113800 | G | A |  |
| Bo7g107550 | 42114019 | G | A |  |
| Bo7g107650 | 42162505 | G | A |  |
| Bo7g107710 | 42201093 | G | A |  |
| Bo7g107710 | 42201174 | G | A |  |
| Bo7g107710 | 42205900 | G | A |  |
| Bo7g107770 | 42239973 | G | A |  |
| Bo7g107770 | 42240132 | G | A |  |
| Bo7g107770 | 42240570 | G | A |  |
| Bo7g107770 | 42240672 | G | A |  |
| Bo7g107770 | 42241236 | G | A |  |
| Bo7g107770 | 42241267 | G | A |  |
| Bo7g107810 | 42275382 | G | A |  |
| Bo7g107810 | 42275673 | G | A |  |
| Bo7g107880 | 42314006 | G | A |  |
| Bo7g107890 | 42317738 | G | A |  |
| Bo7g107910 | 42322907 | G | A |  |
| Bo7g107920 | 42329176 | G | A |  |
| Bo7g107930 | 42331312 | G | A |  |
| Bo7g107930 | 42331369 | G | A |  |
| Bo7g107940 | 42339877 | G | A |  |
| Bo7g107990 | 42377016 | G | A |  |
| Bo7g108200 | 42497033 | G | A |  |
| Bo7g108280 | 42551706 | G | A |  |
| Bo7g108350 | 42582155 | G | A |  |
| Bo7g108380 | 42605123 | G | A |  |
| Bo7g108500 | 42707812 | G | A | SNP_C7_42 |
| Bo7g108630 | 42818433 | G | A |  |
| Bo7g108740 | 42863606 | G | A |  |
| Bo7g108740 | 42863647 | G | A |  |
| Bo7g108740 | 42863773 | G | A | SNP_C7_44 |
| Bo7g108740 | 42863896 | G | A |  |
| Bo7g108790 | 42886693 | G | A |  |
| Bo7g108790 | 42886705 | G | A |  |
| Bo7g108790 | 42886870 | G | A |  |
| Bo7g108790 | 42887711 | G | A |  |
| Bo7g108790 | 42887848 | G | A |  |
| Bo7g108790 | 42887959 | G | A |  |
| Bo7g108790 | 42888001 | G | A | SNP_C7_68 |
| Bo7g108790 | 42888668 | G | A |  |
| Bo7g108790 | 42889606 | G | A |  |
| Bo7g108790 | 42890030 | G | A |  |
| Bo7g108790 | 42890472 | G | A |  |
| Bo7g108810 | 42893427 | G | A |  |
| Bo7g108810 | 42894795 | G | A |  |
| Bo7g108820 | 42908395 | G | A |  |
| Bo7g108820 | 42909195 | G | A |  |
| Bo7g108890 | 42968465 | G | A |  |
| Bo7g108890 | 42968468 | G | A |  |
| Bo7g108940 | 43018797 | G | A |  |
| Bo7g109000 | 43044027 | G | A |  |
| Bo7g109090 | 43093944 | G | A |  |
| Bo7g109090 | 43095273 | G | A |  |
| Bo7g109090 | 43095835 | G | A |  |
| Bo7g109100 | 43111985 | G | A |  |
| Bo7g109120 | 43129394 | G | A |  |
| Bo7g109130 | 43133064 | G | A |  |
| Bo7g109160 | 43147494 | G | A |  |
| Bo7g109260 | 43208376 | G | A |  |
| Bo7g109260 | 43208408 | G | A |  |
| Bo7g109260 | 43208615 | G | A |  |
| Bo7g109270 | 43213517 | G | A |  |
| Bo7g109270 | 43213936 | G | A |  |
| Bo7g109290 | 43235495 | G | A |  |
| Bo7g109340 | 43289273 | G | A |  |
| Bo7g109390 | 43304328 | G | A |  |
| Bo7g109390 | 43304954 | G | A |  |
| Bo7g109420 | 43315214 | G | A |  |
| Bo7g109420 | 43315307 | G | A |  |
| Bo7g109420 | 43315352 | G | A |  |
| Bo7g109420 | 43315388 | G | A |  |
| Bo7g109420 | 43315390 | G | A |  |
| Bo7g109440 | 43322577 | G | A |  |
| Bo7g109460 | 43332853 | G | A |  |
| Bo7g109460 | 43333482 | G | A |  |
| Bo7g109460 | 43334209 | G | A |  |
| Bo7g109490 | 43344889 | G | A |  |
| Bo7g109490 | 43344946 | G | A |  |
| Bo7g109490 | 43345145 | G | A |  |
| Bo7g109490 | 43345160 | G | A |  |
| Bo7g109490 | 43345548 | G | A |  |
| Bo7g109490 | 43345698 | G | A |  |
| Bo7g109500 | 43352412 | G | A |  |
| Bo7g109500 | 43354112 | G | A |  |
| Bo7g109550 | 43373316 | G | A |  |
| Bo7g109550 | 43374668 | G | A |  |
| Bo7g109550 | 43374966 | G | A |  |
| Bo7g109550 | 43375149 | G | A |  |
| Bo7g109570 | 43380709 | G | A |  |
| Bo7g109590 | 43385270 | G | A |  |
| Bo7g109590 | 43385563 | G | A |  |
| Bo7g109590 | 43385594 | G | A |  |
| Bo7g109610 | 43396465 | G | A |  |
| Bo7g109610 | 43399312 | G | A |  |
| Bo7g109610 | 43400032 | G | A |  |
| Bo7g109650 | 43421905 | G | A |  |
| Bo7g109690 | 43450756 | G | A |  |
| Bo7g109690 | 43451170 | G | A |  |
| Bo7g109690 | 43452085 | G | A |  |
| Bo7g109690 | 43452775 | G | A |  |
| Bo7g109700 | 43460304 | G | A |  |
| Bo7g109700 | 43462022 | G | A |  |
| Bo7g109700 | 43462226 | G | A |  |
| Bo7g109710 | 43464148 | G | A |  |
| Bo7g109710 | 43464805 | G | A |  |
| Bo7g109730 | 43469367 | G | A |  |
| Bo7g109750 | 43477860 | G | A |  |
| Bo7g109750 | 43477878 | G | A |  |
| Bo7g109750 | 43478197 | G | A |  |
| Bo7g109750 | 43478226 | G | A |  |
| Bo7g109750 | 43478778 | G | A |  |
| Bo7g109760 | 43485215 | G | A |  |
| Bo7g109760 | 43485851 | G | A |  |
| Bo7g109760 | 43486223 | G | A |  |
| Bo7g109760 | 43486487 | G | A |  |
| Bo7g109760 | 43486596 | G | A |  |
| Bo7g109760 | 43487539 | G | A |  |
| Bo7g109760 | 43487614 | G | A |  |
| Bo7g109770 | 43488878 | G | A |  |
| Bo7g109800 | 43497147 | G | A |  |
| Bo7g109800 | 43497177 | G | A |  |
| Bo7g109800 | 43498028 | G | A |  |
| Bo7g109800 | 43498325 | G | A |  |
| Bo7g109800 | 43498737 | G | A |  |
| Bo7g109800 | 43498749 | G | A |  |
| Bo7g109800 | 43498809 | G | A |  |
| Bo7g109810 | 43501202 | G | A |  |
| Bo7g109810 | 43502830 | G | A |  |
| Bo7g109810 | 43504023 | G | A |  |
| Bo7g109820 | 43505170 | G | A |  |
| Bo7g109820 | 43506158 | G | A |  |
| Bo7g109850 | 43513351 | G | A |  |
| Bo7g109850 | 43513795 | G | A |  |
| Bo7g109900 | 43532359 | G | A |  |
| Bo7g109900 | 43532900 | G | A |  |
| Bo7g109920 | 43537941 | G | A |  |
| Bo7g109920 | 43537956 | G | A |  |
| Bo7g109930 | 43539121 | G | A |  |
| Bo7g109950 | 43547884 | G | A |  |
| Bo7g109990 | 43561259 | G | A |  |
| Bo7g110190 | 43638455 | G | A |  |
| Bo7g110190 | 43638464 | G | A |  |
| Bo7g110190 | 43638503 | G | A |  |
| Bo7g110220 | 43651045 | G | A |  |
| Bo7g110260 | 43670135 | G | A |  |
| Bo7g110470 | 43803617 | G | A |  |
| Bo7g110470 | 43804634 | G | A |  |
| Bo7g110500 | 43818000 | G | A |  |
| Bo7g110500 | 43818102 | G | A |  |
| Bo7g110500 | 43818111 | G | A |  |
| Bo7g110500 | 43819702 | G | A |  |
| Bo7g110500 | 43820577 | G | A |  |
| Bo7g110500 | 43820787 | G | A |  |
| Bo7g110560 | 43849149 | G | A |  |
| Bo7g110580 | 43856285 | G | A |  |
| Bo7g110580 | 43856288 | G | A |  |
| Bo7g110580 | 43857096 | G | A |  |
| Bo7g110630 | 43906480 | G | A |  |
| Bo7g106020 | 41037385 | T | A |  |
| Bo7g106250 | 41179067 | T | A |  |
| Bo7g106470 | 41314323 | T | A |  |
| Bo7g106490 | 41329836 | T | A |  |
| Bo7g106820 | 41623357 | T | A |  |
| Bo7g106960 | 41697068 | T | A |  |
| Bo7g106960 | 41697535 | T | A |  |
| Bo7g106960 | 41697607 | T | A |  |
| Bo7g107080 | 41810871 | T | A |  |
| Bo7g107100 | 41842784 | T | A |  |
| Bo7g107100 | 41843870 | T | A |  |
| Bo7g107100 | 41844144 | T | A |  |
| Bo7g107100 | 41844669 | T | A |  |
| Bo7g107100 | 41845301 | T | A |  |
| Bo7g107190 | 41880109 | T | A |  |
| Bo7g107190 | 41888530 | T | A |  |
| Bo7g107330 | 41935743 | T | A |  |
| Bo7g107410 | 41993867 | T | A |  |
| Bo7g107510 | 42086655 | T | A |  |
| Bo7g107550 | 42111446 | T | A |  |
| Bo7g107550 | 42111773 | T | A |  |
| Bo7g107550 | 42113695 | T | A |  |
| Bo7g107710 | 42200846 | T | A |  |
| Bo7g107710 | 42206163 | T | A |  |
| Bo7g107890 | 42317187 | T | A |  |
| Bo7g107890 | 42317798 | T | A |  |
| Bo7g107910 | 42321882 | T | A |  |
| Bo7g107910 | 42322322 | T | A |  |
| Bo7g107920 | 42328843 | T | A |  |
| Bo7g107930 | 42331318 | T | A |  |
| Bo7g108280 | 42550933 | T | A |  |
| Bo7g108340 | 42579250 | T | A |  |
| Bo7g108350 | 42581808 | T | A |  |
| Bo7g108500 | 42706821 | T | A |  |
| Bo7g108570 | 42792948 | T | A |  |
| Bo7g108740 | 42863642 | T | A |  |
| Bo7g108790 | 42887917 | T | A |  |
| Bo7g108790 | 42889591 | T | A |  |
| Bo7g108790 | 42890481 | T | A |  |
| Bo7g108810 | 42893835 | T | A |  |
| Bo7g108930 | 43016164 | T | A |  |
| Bo7g109090 | 43094455 | T | A |  |
| Bo7g109210 | 43165631 | T | A |  |
| Bo7g109250 | 43196839 | T | A |  |
| Bo7g109260 | 43208757 | T | A |  |
| Bo7g109290 | 43235919 | T | A |  |
| Bo7g109290 | 43236267 | T | A |  |
| Bo7g109350 | 43292825 | T | A |  |
| Bo7g109350 | 43293696 | T | A |  |
| Bo7g109420 | 43315231 | T | A |  |
| Bo7g109420 | 43315411 | T | A |  |
| Bo7g109430 | 43319803 | T | A |  |
| Bo7g109460 | 43332715 | T | A |  |
| Bo7g109500 | 43352111 | T | A |  |
| Bo7g109500 | 43353897 | T | A |  |
| Bo7g109610 | 43400769 | T | A |  |
| Bo7g109650 | 43421095 | T | A |  |
| Bo7g109650 | 43421137 | T | A |  |
| Bo7g109690 | 43450024 | T | A |  |
| Bo7g109690 | 43452264 | T | A |  |
| Bo7g109700 | 43457573 | T | A |  |
| Bo7g109700 | 43459885 | T | A |  |
| Bo7g109710 | 43464608 | T | A |  |
| Bo7g109730 | 43468709 | T | A |  |
| Bo7g109760 | 43485671 | T | A |  |
| Bo7g109800 | 43497900 | T | A |  |
| Bo7g109800 | 43497921 | T | A |  |
| Bo7g109800 | 43498875 | T | A |  |
| Bo7g109840 | 43510953 | T | A |  |
| Bo7g109950 | 43546715 | T | A |  |
| Bo7g109950 | 43547850 | T | A |  |
| Bo7g109990 | 43566462 | T | A |  |
| Bo7g110260 | 43670032 | T | A |  |
| Bo7g110500 | 43817691 | T | A |  |
| Bo7g110500 | 43819207 | T | A |  |
| Bo7g110500 | 43819675 | T | A |  |
| Bo7g110560 | 43848794 | T | A |  |
| Bo7g110580 | 43856216 | T | A |  |
| Bo7g110580 | 43857081 | T | A |  |

**Table S4. Gene annotation in the *Rcr7* target region using Blast2g**

| SeqName | Length | Description in Blast2go |
| --- | --- | --- |
| Bo7g108740 | 864 | plasma membrane intrinsic 1B [Arabidopsis thaliana] |
| Bo7g108750 | 846 | senescence-associated -like [Arabidopsis thaliana] |
| Bo7g108760 | 2724 | Disease resistance (TIR-NBS class) [Arabidopsis thaliana] |
| Bo7g108770 | 477 | RING U-box superfamily [Arabidopsis thaliana] |
| Bo7g108780 | 771 | F-box family [Arabidopsis thaliana] |
| Bo7g108790 | 2643 | Adaptin family [Arabidopsis thaliana] |
| Bo7g108800 | 732 | PLAC8 family [Arabidopsis thaliana] |
| Bo7g108810 | 1533 | transferring glycosyl group transferase (DUF604) [Arabidopsis thaliana] |
| Bo7g108820 | 1482 | Pectin lyase-like superfamily [Arabidopsis thaliana] |
| Bo7g108830 | 1032 | Toll-Interleukin-Resistance (TIR) domain family [Arabidopsis thaliana] |
| Bo7g108840 | 414 | Toll-Interleukin-Resistance (TIR) domain family [Arabidopsis thaliana] |
| Bo7g108850 | 789 | Toll-Interleukin-Resistance (TIR) domain family [Arabidopsis thaliana] |
| Bo7g108860 | 1128 | (DUF793) [Arabidopsis thaliana] |
| Bo7g108870 | 900 | Disease resistance (TIR-NBS-LRR class) [Arabidopsis thaliana] |
| Bo7g108880 | 1443 | cellulase [Arabidopsis thaliana] |
| Bo7g108890 | 1032 | phosphatase [Arabidopsis thaliana] |
| Bo7g108900 | 483 | hAT family dimerization domain-containing [Arabidopsis thaliana] |
| Bo7g108910 | 1248 | Tyrosine transaminase family [Arabidopsis thaliana] |
| Bo7g108920 | 1224 | Tyrosine transaminase family [Arabidopsis thaliana] |
| Bo7g108930 | 807 | Reticulon family [Arabidopsis thaliana] |
| Bo7g108940 | 2973 | calcium-dependent kinase [Arabidopsis thaliana] |
| Bo7g108950 | 729 | partial [Arabidopsis thaliana] |
| Bo7g108960 | 213 | Polyketide cyclase dehydrase and lipid transport superfamily [Arabidopsis thaliana] |
| Bo7g108970 | 606 | non-LTR retroelement reverse transcriptase |
| Bo7g108980 | 456 | Polyketide cyclase dehydrase and lipid transport superfamily [Arabidopsis thaliana] |
| Bo7g108990 | 351 | retroelement pol poly -like |
| Bo7g109000 | 564 | Disease resistance-responsive (dirigent ) family [Arabidopsis thaliana] |
| Bo7g109010 | 444 | RNA-directed DNA polymerase (reverse transcriptase)-related family [Arabidopsis thaliana] |
| Bo7g109020 | 2472 | cation H+ exchanger 18 [Arabidopsis thaliana] |
| Bo7g109030 | 333 | vacuolar membrane ATPase 10 [Arabidopsis thaliana] |
| Bo7g109040 | 189 | Ribosomal S30 family [Arabidopsis thaliana] |
| Bo7g109050 | 183 | GRF zinc finger [Arabidopsis thaliana] |
| Bo7g109060 | 948 | transmembrane (DUF1191) [Arabidopsis thaliana] |
| Bo7g109070 | 3009 | Disease resistance (TIR-NBS-LRR class) family [Arabidopsis thaliana] |
| Bo7g109080 | 918 | Galactose mutarotase-like superfamily [Arabidopsis thaliana] |
| Bo7g109090 | 1848 | Leucine-rich repeat kinase family [Arabidopsis thaliana] |


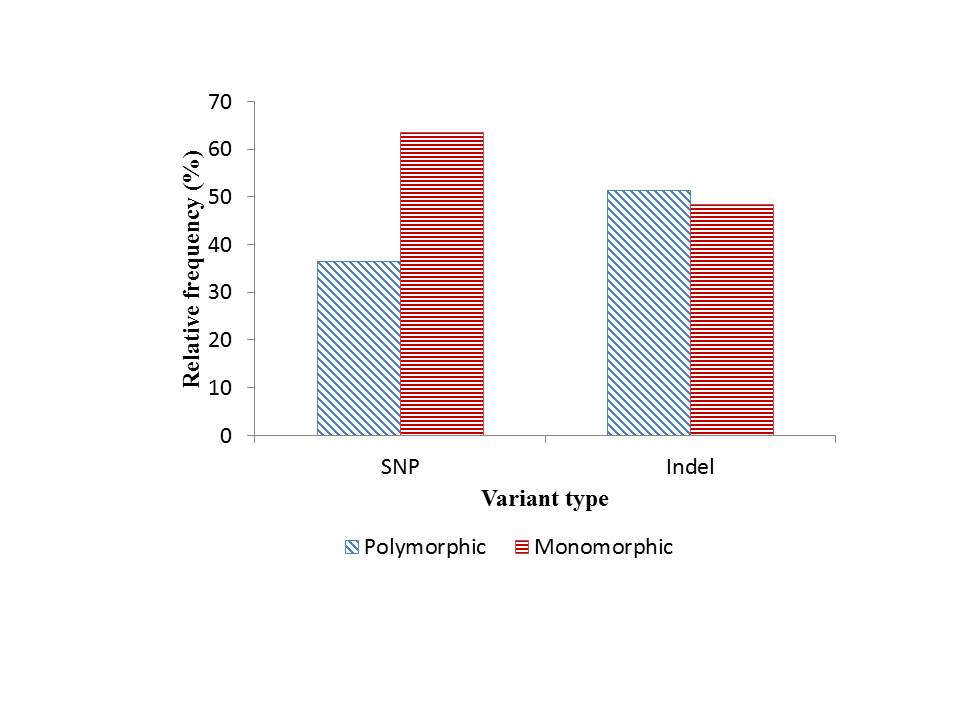


**Fig. S1,**  **Percentages of SNPs and InDels found in the R and S pools.**


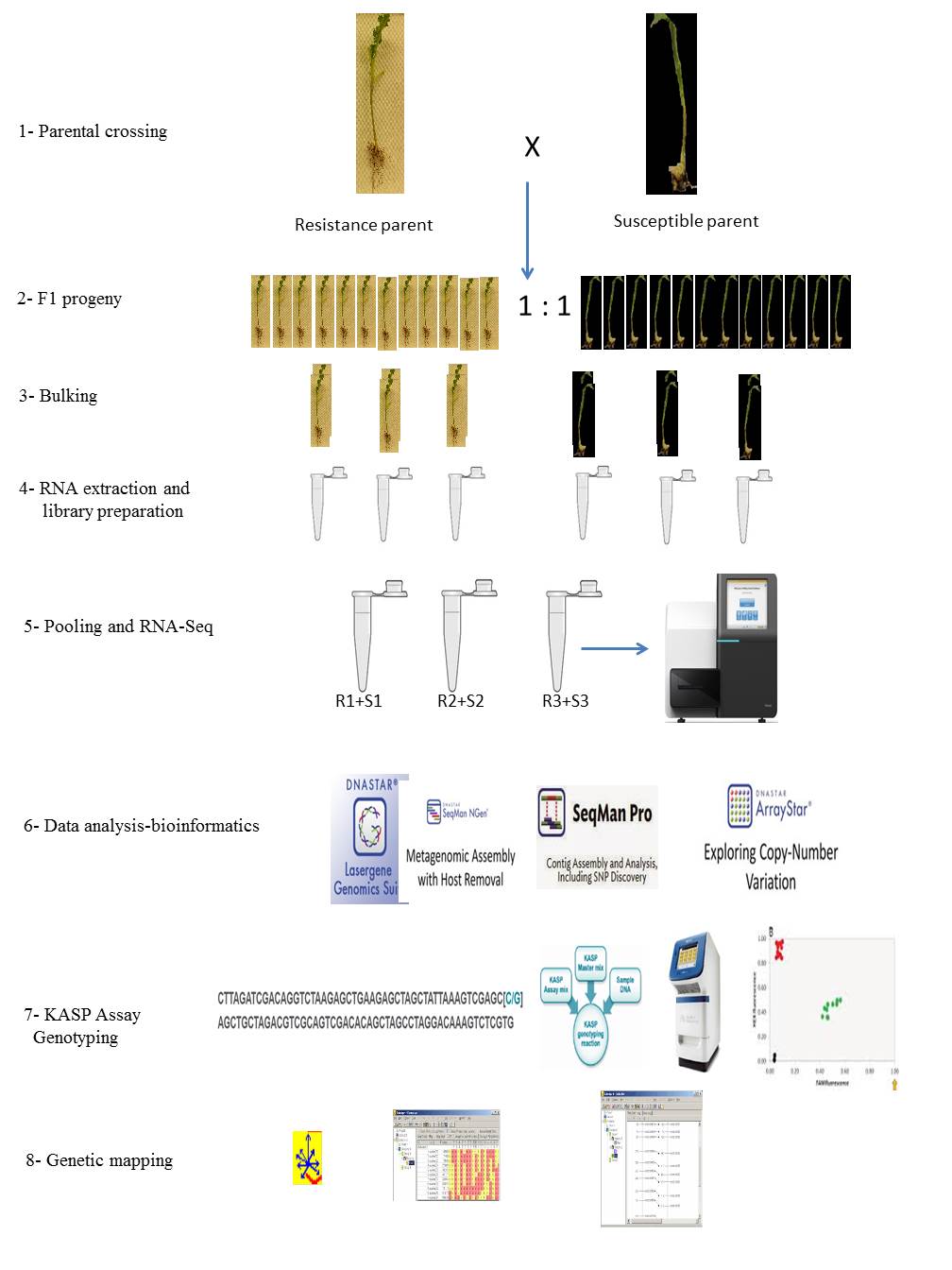


**Fig. S2**. **Schematic flowchart of the experimental procedure**. Pictures represent the instruments and software used.

(a) Cross pollination between the resistant parent (‘Tekila’) and the susceptible parent T010000 (DH3); (b) F_1_ generation with a 1:1 segregation ratio; (c) Bulking: based on their responses to clubroot, each bulk of the progeny (resistant and susceptible) was divided into three bulks that each consisted of 30 plants; (d) RNA extraction and library preparation were done on individual bulks (six libraries); (e) Pooling and sequencing: the library of one resistant bulks and one susceptible one were pooled together, resulting in three pooled libraries, and RNA sequencing was done for each pooled library; (f) Data analysis of the sequence assembly was done separately for the resistant and susceptible pools. Each pool comprised of the sequence reads from the three bulks; (g) a Kasp assay was developed for each set of SNP primers and polymorphic SNP markers were used to genotype the segregating population; (h) SNP markers closely linked to the gene *Rcr7* were mapped to chromosome C7 of *B. oleracea*.
